# Assessment of symptoms in COMET-ICE, a phase 2/3 study of sotrovimab for early treatment of non-hospitalized patients with COVID-19

**DOI:** 10.1186/s41687-023-00621-8

**Published:** 2023-09-13

**Authors:** Sacha Satram, Parima Ghafoori, Carolina M. Reyes, Tom J. H. Keeley, Helen J. Birch, Dimitra Brintziki, Melissa Aldinger, Elizabeth Alexander, Amanda Lopuski, Elias H. Sarkis, Anil Gupta, Adrienne E. Shapiro, John H. Powers

**Affiliations:** 1 Vir Biotechnology, Inc., San Francisco, CA USA; 2 GSK, Collegeville, PA USA; 3 GSK, Brentford, Middlesex, UK; 4 Sarkis Clinical Trials, Gainesville, FL USA; 5 Albion Finch Medical, William Osler Health Centre, Toronto, ON Canada; 6 Departments of Global Health and Medicine, University of Washington and Fred Hutchinson Cancer Center, Seattle, WA USA; 7 George Washington University School of Medicine, Washington, DC USA

**Keywords:** COVID-19, FLU-PRO Plus, Monoclonal antibody, Patient-reported outcomes, PRO, Sotrovimab, Symptoms

## Abstract

**Background:**

The COMET-ICE trial demonstrated that sotrovimab clinically and statistically significantly reduces the risk of all-cause > 24-h hospitalization or death due to any cause among patients with COVID-19 at high risk of disease progression. Patient-reported outcomes are important to capture symptom burden of COVID-19 and assess treatment effectiveness. This study investigated symptoms and their impact over the acute phase of COVID-19 infection among patients on sotrovimab versus placebo.

**Methods:**

Randomized (1:1), double-blind, multicenter, placebo-controlled, phase 2/3 study in 57 centers across five countries. Participants were non-hospitalized patients with symptomatic, mild-to-moderate COVID-19 and ≥ 1 baseline risk factor for disease progression (aged ≥ 55 years or ≥ 1 of the following: diabetes requiring medication, obesity, chronic kidney disease, congestive heart failure, chronic obstructive pulmonary disease, or moderate-to-severe asthma). An intravenous infusion of sotrovimab 500 mg or placebo was administered on Day 1. The FLU-PRO Plus questionnaire was administered once-daily with 24-h recall from Day 1–21, and at Day 29. Intensity and duration of COVID-19 symptoms were determined from area under the curve (AUC) and mean change in total and individual domain scores through Days 7, 14, and 21. Time to symptom alleviation was assessed.

**Results:**

In total, 1057 patients were randomized to sotrovimab (n = 528) or placebo (n = 529). At Day 7, mean decrease in FLU-PRO Plus total score (measured by AUC) was statistically significantly greater for patients on sotrovimab (–3.05 [95% confidence interval (CI) –3.27 to –2.83]) than placebo (–1.98 [95% CI –2.20 to –1.76]; difference –1.07 [95% CI –1.38 to –0.76]; *p* < 0.001). Significant differences were also observed at Days 14 and 21. A more rapid decline in symptom severity was observed with sotrovimab versus placebo through Week 1 and the first 21 days post-treatment. By Day 21, 41% of patients on sotrovimab and 34% on placebo reported symptom resolution. In a post-hoc analysis, median time to symptom alleviation was 4 and 6 days, respectively.

**Conclusions:**

Sotrovimab provides significant and rapid improvements in patient-reported COVID-19 symptoms, as measured by the FLU-PRO Plus. These results further show the benefits of sotrovimab in alleviating symptoms among high-risk patients with COVID-19.

*Trial registration* ClinicalTrials.Gov: NCT04545060 (https://clinicaltrials.gov/ct2/show/NCT04545060). Date of registration: September 10, 2020 (retrospectively registered).

**Supplementary Information:**

The online version contains supplementary material available at 10.1186/s41687-023-00621-8.

## Introduction

Sotrovimab is an Fc-engineered pan-sarbecovirus human monoclonal antibody, which was developed for the treatment of COVID-19 from a parental antibody isolated from a survivor of the severe acute respiratory syndrome (SARS) outbreak in 2003 [1–5]. It targets a conserved epitope in the SARS-CoV-2 spike protein, and preclinical studies have shown that sotrovimab retains neutralizing activity against variants of concern that were circulating during the time of enrolment for this trial [5].

The **CO**vid-19 **M**onoclonal antibody **E**fficacy **T**rial-**I**ntent to **C**are **E**arly (COMET-ICE; NCT04545060) trial evaluated the efficacy and safety of sotrovimab administered intravenously in non-hospitalized patients with mild to moderate COVID-19 at high risk of progression to severe disease (n = 1057). All-cause > 24-h hospitalization or death due to any case was significantly reduced with sotrovimab (6/528; 1%) compared with placebo (30/529; 6%) at Day 29 (*p* < 0.001; adjusted relative risk, 0.21 [95% confidence interval (CI) 0.09–0.50]) [6].

Globally, COVID-19 is associated with significant clinical, public health and economic burden, leading to missed school and work to recover from illness [7, 8]. Patient-reported outcomes (PROs) are important in order to fully capture the symptom burden of COVID-19 and assess the effectiveness of treatments [9]. Epidemiological studies show viral respiratory diseases share similar symptom profiles, including fever/chills, cough, shortness of breath, fatigue, sore throat, muscle pain or body aches, headache, vomiting, and diarrhea [10]. The inFLUenza Patient-Reported Outcome (FLU-PRO) measure was developed to assess the core symptoms of influenza and other viral respiratory diseases [11]. An extended version of the FLU-PRO, called the FLU-PRO Plus, has subsequently been developed to include additional commonly reported COVID-19 symptoms of loss of taste and smell [12]. The content validity and psychometric properties of the FLU-PRO Plus in patients with COVID-19 have been recently reported [13]. Concepts measured were shown to be relevant and important to patients with COVID-19, and questions/responses were comprehensive and understandable. Psychometric analyses supported the reliability, validity, and responsiveness of the FLU-PRO Plus in individuals with symptoms of COVID-19.

In COMET-ICE, the FLU-PRO Plus questionnaire was used to assess symptoms and impacts of COVID-19, along with the Short Form-12 (SF-12) Hybrid questionnaire (SF-12 plus the full SF-36 domains of vitality and physical role) to measure self-reported functional health and wellbeing, and the validated, patient-reported Work Productivity and Activity Impairment (WPAI) tool to quantitively assess absenteeism, presenteeism, work productivity loss, and activity impairment. Here we report the results for these PRO assessments in the COMET-ICE trial up to Day 29.

## Methods

COMET-ICE was a randomized, double-blind, multicenter, placebo-controlled phase 2/3 study, involving 57 centers across five countries (CONSORT-PRO checklist available in Additional file 1). The study design has been previously reported in detail [6]. Eligible participants were aged ≥ 18 years old, had tested positive for SARS-CoV-2 by reverse transcription polymerase chain reaction (RT-PCR) or antigen test, had oxygen saturation of ≥ 94% on room air, and had symptom onset within the previous 5 days. Patients had to be considered at high risk for COVID-19 progression to hospitalization or death because of older age (≥ 55 years) or due to the presence of at least one of the following risk factors: diabetes requiring medication, obesity (body mass index [BMI] > 30 kg/m^2^ [original protocol]; > 35 kg/m^2^ [protocol amendment 1]), chronic kidney disease (glomerular filtration rate < 60 mL/min/1.73 m^2^), congestive heart failure (New York Heart Association class II or higher), chronic obstructive pulmonary disease, or moderate-to-severe asthma. Patients enrolled between August 2020 and March 2021 were randomized (1:1, n = 1057) to a single intravenous infusion of sotrovimab 500 mg (n = 528) or placebo (n = 529) and followed for 24 weeks (flow diagram published previously [6]).

The COMET-ICE study was conducted in accordance with the principles of the Declaration of Helsinki and Council for International Organizations of Medical Sciences International Ethical Guidelines, applicable International Council for Harmonisation Good Clinical Practice guidelines, and applicable laws and regulations. Written informed consent was provided by all patients prior to study entry.

### PRO measurements

Questionnaires were administered using an electronic device where possible. A paper PRO option was used in instances where logistical or technical difficulties in using electronic PROs were experienced. FLU-PRO Plus was completed once-daily with a 24-h recall, from Day 1 through Day 21, and then at Day 29, Week 8, and Week 12. Data through Day 29 are reported in this manuscript.

COVID-19 symptoms were assessed across the 32 FLU-PRO Plus items and predominantly scored on a five-point severity scale (higher scores indicating more severe symptoms). Severity and duration of symptoms were quantified using the averaged change from baseline in FLU-PRO Plus score (as measured by area under the curve [AUC]) through Day 7, and through Day 14 and Day 21. FLU-PRO Plus total score did not include taste and smell assessments (i.e., sense domain) and these were instead accounted for as a separate binary score; individual domains were nose, throat, eyes, chest/respiratory, gastrointestinal, body/systemic, and sense.

Subgroup analyses by symptom severity at baseline were performed for the FLU-PRO Plus endpoints among patients with ≥ 2 symptoms of moderate/higher intensity and those with < 2 symptoms of moderate/higher intensity.

Time to sustained (≥ 48 h) symptom alleviation (i.e., responder definition), was measured by the FLU-PRO Plus over the first 21 days. Sustained (≥ 48 h) symptom resolution was defined as the absence of the majority of core symptoms of COVID-19 except for cough and fatigue items scoring no more than “Somewhat” in severity, but loss of smell or taste allowed; this definition was developed in line with the U.S. Food and Drug Administration (FDA) draft guidance [14]. Full details of the responder definition(s) used are included in the supplemental information. A participant could only be classified as having achieved sustained symptom alleviation if they had two or more, non-missing, consecutively scored questionnaires that showed symptom alleviation. Those who never achieved sustained symptom alleviation or did not complete the questionnaires were then censored at day of withdrawal or Day 21, whatever was earliest. Additionally, a post-hoc sensitivity analysis of time to symptom alleviation was conducted using the FLU-PRO user manual definition (total score ≤ 1 and all domain scores ≤ 1, excluding the sense domain). The proportion of patients achieving sustained symptom alleviation through Day 21 was also described.

Health-related quality of life was measured by the SF-12 Hybrid, an established measure of health-related quality of life which has demonstrated validity in many disease areas [15–17], in order to assess COVID-19 impacts on quality of life. The SF-12 Hybrid was completed (with 24-h recall) on Day 1, Day 15, and Day 29. Starting at Week 8, participants completed the questionnaire monthly through Week 24. The WPAI was also completed on Day 1, Day 15, and Day 29. Starting at Week 8, participants completed the questionnaire monthly. A higher score in both assessment tools is indicative of better quality of life. As above, only data through Day 29 are included in this manuscript.

### Statistical analysis

Change in FLU-PRO Plus total score (AUC) through Day 7 was a secondary trial endpoint, formally tested with an alpha level of 5% (two-sided) after adjustment for multiplicity using a pre-specified testing hierarchy (Fig. S1 in Additional file 2). Change in FLU-PRO Plus total and domain scores (AUC) through Day 14 and 21 were exploratory trial endpoints. The FLU-PRO Plus endpoints were evaluated using analyses of covariance adjusted for treatment, baseline value, age group (≤ 70 years versus > 70 years), duration of symptoms group (≤ 3 days versus ≥ 4 days), sex, and region. A post-hoc analysis to calculate Cohen’s d effect size for the average change in FLU-PRO Plus total score (AUC) analysis was conducted.

Subgroup analyses by symptom severity were adjusted for treatment, baseline value, age group, duration of symptoms group, sex, and region, along with a treatment by subgroup interaction term.

Missing data, which were mostly due to challenges with electronic questionnaire software, failure to complete paper questionnaires, or lost-to-follow-up data, were imputed using a modified last-observation carried forward approach for the final assessment only (i.e., Day 7, Day 14, or Day 21). If a FLU-PRO score was missing, the last non-missing, post-baseline score from that week (i.e., Day 2–6 for missing Day 7, Day 8–13 for missing Day 14, and Day 15–20 for missing Day 21) was carried forward as the Day 7/14/21 score. If no non-missing score was available, no score was imputed and the AUC was not calculated.

Time to sustained (≥ 48 h) symptom alleviation was analyzed using the Kaplan–Meier methods and a log-rank test stratified by region, duration of symptoms group, age group, and sex.

For the exploratory endpoints of WPAI and SF-12 Hybrid, mean change in scores were summarized and no formal statistical analysis was conducted.

## Results

Completed FLU-PRO Plus questionnaires were available from 84% of participants in the sotrovimab arm (n = 446) and 83% of participants in the placebo arm (n = 437) on Day 1. By Day 21, completed questionnaires were available for 50% of participants in the sotrovimab arm (n = 262) and 48% of participants in the placebo arm (n = 250). Full details of questionnaire availability and proportions of electronic PROs versus paper questionnaires are shown in Table S1 in Additional file 2. In a post-hoc analysis, patient demographic and baseline disease characteristics were compared among patients with missing data versus those without. We did not find any notable differences between these two populations.

### FLU-PRO Plus total and domain scores

Mean decrease in FLU-PRO Plus total score as measured by the AUC_0-7_ was statistically significantly greater in the sotrovimab arm (–3.05 [95% CI –3.27 to –2.83]) than in the placebo arm (–1.98 [95% CI –2.20 to –1.76]; difference –1.07 [95% CI –1.38 to –0.76]; *p* < 0.001; corresponding to a moderate statistical effect size [Cohen’s d 0.48]) at Day 7 [6] (secondary endpoint, part of testing hierarchy, Table 1). Differences between the treatment arms were also statistically significant up to Day 14 (–2.35 [95% CI –3.00 to –1.70]; *p* < 0.001; corresponding to a moderate statistical effect size [Cohen’s d 0.52]) and Day 21 (–3.09 [95% CI –4.05 to –2.12]; *p* < 0.001; corresponding to a moderate statistical effect size [Cohen’s d 0.47]) (exploratory endpoints, Table 1). The difference between treated and placebo groups increased over time. Results for individual domains of the FLU-PRO Plus were consistent with those for total score; mean decreases were statistically significantly greater with sotrovimab versus placebo up to Day 7, 14, and 21 (Table 1). The change from baseline in FLU-PRO Plus total and domain scores was also analyzed and results were consistent with those of the AUC analysis (Table S2 in Additional file 2).

**Table 1:** Average change from baseline (AUC) of COVID-19-related illness as measured by FLU-PRO Plus (total and domain scores) for overall population

	Overall population	
	Placebo (N = 529)	Sotrovimab (500 mg IV) (N = 528)
*Total*		
AUC to Day 7		
n	399	412
Mean (95% CI)	− 1.98 (− 2.20 to − 1.76)	− 3.05 (− 3.27 to − 2.83)
Difference (95% CI)	− 1.07 (− 1.38 to − 0.76)	
* p* value	< 0.001	
AUC to Day 14		
n	373	385
Mean (95% CI)	− 7.04 (− 7.51 to − 6.58)	− 9.40 (− 9.85 to − 8.94)
Difference (95% CI)	− 2.35 (− 3.00 to − 1.70)	
*p* value	< 0.001	
AUC to Day 21		
n	345	379
Mean (95% CI)	− 13.34 (− 14.03 to − 12.64)	− 16.42 (− 17.09 to − 15.76)
Difference (95% CI)	− 3.09 (− 4.05 to − 2.12)	
*p* value	< 0.001	
*Nose*		
AUC to Day 7		
n	401	412
Mean (95% CI)	− 2.20 (− 2.49 to − 1.91)	− 3.06 (− 3.34 to − 2.77)
Difference (95% CI)	− 0.86 (− 1.26 to − 0.45)	
*p* value	< 0.001	
AUC to Day 14		
n	375	385
Mean (95% CI)	− 7.90 (− 8.48 to − 7.33)	− 9.68 (− 10.25 to − 9.11)
Difference (95% CI)	− 1.78 (− 2.59 to − 0.97)	
*p* value	< 0.001	
AUC to Day 21		
n	347	379
Mean (95% CI)	− 14.73 (− 15.58 to − 13.87)	− 16.95 (− 17.77 to − 16.13)
Difference (95% CI)	− 2.22 (− 3.41 to − 1.04)	
*p* value	< 0.001	
*Throat*		
AUC to Day 7		
n	401	412
Mean (95% CI)	− 2.16 (− 2.44 to − 1.87)	− 3.03 (− 3.31 to − 2.75)
Difference (95% CI)	− 0.88 (− 1.28 to − 0.48)	
*p* value	< 0.001	
AUC to Day 14		
n	375	385
Mean (95% CI)	− 6.99 (− 7.54 to − 6.44)	− 8.97 (− 9.51 to − 8.42)
Difference (95% CI)	− 1.98 (− 2.75 to − 1.21)	
*p* value	< 0.001	
AUC to Day 21		
n	347	379
Mean (95% CI)	− 12.78 (− 13.58 to − 11.98)	− 15.35 (− 16.11 to − 14.58)
Difference (95% CI)	− 2.57 (− 3.68 to − 1.46)	
*p* value	< 0.001	
*Eyes*		
AUC to Day 7		
n	401	412
Mean (95% CI)	− 1.54 (− 1.81 to − 1.27)	− 2.33 (− 2.59 to − 2.06)
Difference (95% CI)	− 0.79 (− 1.17 to − 0.41)	
*p* value	< 0.001	
AUC to Day 14		
n	375	385
Mean (95% CI)	− 5.29 (− 5.82 to − 4.76)	− 7.03 (− 7.56 to− 6.51)
Difference (95% CI)	− 1.74 (− 2.49 to − 0.99)	
*p* value	< 0.001	
AUC to Day 21		
n	347	379
Mean (95% CI)	− 9.76 (− 10.56 to − 8.95)	− 12.07 (− 12.83 to − 11.30)
Difference (95% CI)	− 2.31 (− 3.42 to − 1.20)	
*p* value	< 0.001	
*Chest/respiratory*		
AUC to Day 7		
n	401	412
Mean (95% CI)	− 1.30 (− 1.56 to − 1.04)	− 2.60 (− 2.86 to − 2.35)
Difference (95% CI)	− 1.30 (− 1.66 to − 0.93)	
*p* value	< 0.001	
AUC to Day 14		
n	375	385
Mean (95% CI)	− 5.45 (− 6.02 to − 4.88)	− 8.42 (–8.98 to − 7.85)
Difference (95% CI)	− 2.97 (− 3.77 to − 2.17)	
*p* value	< 0.001	
AUC to Day 21		
n	347	379
Mean (95% CI)	− 11.05 (− 11.91 to − 10.19)	− 15.27 (− 16.09 to − 14.44)
Difference (95% CI)	− 4.22 (− 5.41 to − 3.02)	
* p* value	< 0.001	
*Gastrointestinal*		
AUC to Day 7		
n	399	412
Mean (95% CI)	− 1.47 (− 1.69 to − 1.25)	− 2.15 (− 2.36 to − 1.93)
Difference (95% CI)	− 0.68 (− 0.98 to − 0.37)	
*p* value	< 0.001	
AUC to Day 14		
n	373	385
Mean (95% CI)	− 4.88 (− 5.30 to − 4.47)	− 6.55 (− 6.95 to − 6.15)
Difference (95% CI)	− 1.66 (− 2.24 to − 1.09)	
* p* value	< 0.001	
AUC to Day 21		
n	345	379
Mean (95% CI)	− 9.17 (− 9.76 to − 8.58)	− 11.03 (− 11.59 to − 10.47)
Difference (95% CI)	− 1.86 (− 2.67 to − 1.05)	
*p* value	< 0.001	
*Body/systemic*		
AUC to Day 7		
n	400	412
Mean (95% CI)	− 2.55 (− 2.83 to − 2.27)	− 3.88 (− 4.16 to − 3.60)
Difference (95% CI)	− 1.33 (− 1.73 to − 0.93)	
*p* value	< 0.001	
AUC to Day 14		
n	374	385
Mean (95% CI)	− 8.98 (− 9.55 to − 8.41)	− 11.75 (− 12.31 to − 11.18)
Difference (95% CI)	− 2.76 (− 3.57 to − 1.96)	
*p* value	< 0.001	
AUC to Day 21		
n	346	379
Mean (95% CI)	− 16.87 (− 17.71 to − 16.03)	− 20.45 (− 21.26 to − 19.65)
Difference (95% CI)	− 3.59 (− 4.75 to − 2.42)	
*p* value	< 0.001	
*Sense*		
AUC to Day 7		
n	399	412
Mean (95% CI)	− 0.10 (− 0.28 to − 0.07)	− 0.55 (− 0.73 to − 0.38)
Difference (95% CI)	− 0.45 (− 0.70 to − 0.21)	
*p* value	< 0.001	
AUC to Day 14		
n	373	385
Mean (95% CI)	− 1.20 (− 1.60 to − 0.79)	− 2.39 (− 2.79 to − 1.99)
Difference (95% CI)	−1.19 (−1.76 to −0.62)	
*p* value	< 0.001	
AUC to Day 21		
n	345	379
Mean (95% CI)	− 3.32 (− 3.95 to − 2.68)	− 4.85 (− 5.46 to − 4.24)
Difference (95% CI)	− 1.53 (− 2.41 to − 0.65)	
*p* value	< 0.001	

Total score analysis through Day 7 is a secondary endpoint, part of the testing hierarchy. Analysis through Day 14 and 21 (total and domain scores) are exploratory endpoints. Analysis was performed using an ANCOVA model, adjusting for region (Europe, North America, South America), duration of symptoms (≤ 3 days versus ≥ 4 days), age (≤ 70 versus > 70 years), sex (male, female), and baseline score

*ANCOVA* analysis of covariance, *AUC* area under the curve, *CI* confidence interval, *FLU-PRO Plus* inFLUenza Patient-Reported Outcome Plus, *IV* intravenous

**Fig. 1:**
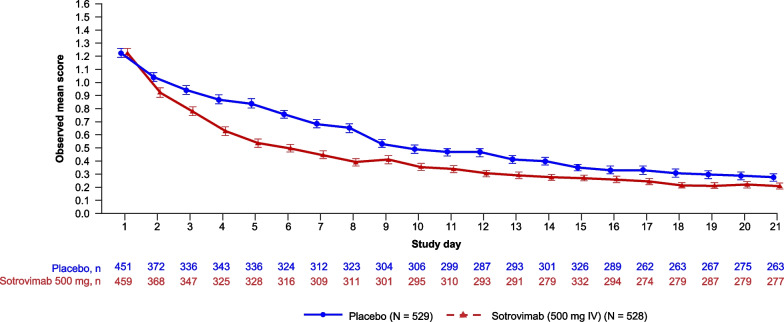
Observed mean daily FLU-PRO Plus total score by study day. Based on data through Day 21, as shown in the end of study Week 24 database. *FLU-PRO Plus* inFLUenza Patient-Reported Outcome Plus, *IV* intravenous

Figure 1 shows the observed mean daily FLU-PRO Plus total score based on available data. Consistent with the AUC results, we observed a more rapid decline in symptom severity in the sotrovimab arm compared with placebo within the first week and throughout the first 21 days after treatment. Observed mean daily scores for the individual domains were generally consistent with those for total score (Figs. S2–8 in Additional file 2).

### FLU-PRO Plus total and domain score subgroup analyses

Results of the FLU-PRO Plus subgroup analyses of mean change from baseline in total score (AUC) were generally consistent with those for the overall population (Table S3 in Additional file 2). For participants with < 2 moderate/higher symptom severity at baseline (sotrovimab: n = 234; placebo: n = 231), mean decrease in total score of FLU-PRO Plus (AUC) was greater in the sotrovimab arm than the placebo arm at Day 7, 14, and 21 (difference at Day 7: –1.22; Day 14: –2.59; Day 21: –3.29, all *p* < 0.001; Table S3 in Additional file 2). Results for the mean decrease in each domain score (AUC) were consistent with total score, showing that improvements were consistent in all aspects of disease and not driven by the effects of one or a few domains. For participants with ≥ 2 moderate/higher symptom severity at baseline (sotrovimab: n = 212; placebo: n = 206), the mean decrease in total score of FLU-PRO Plus (AUC) was greater in the sotrovimab arm than the placebo arm at Days 7, 14, and 21 (difference at Day 7: –0.74; Day 14: –1.65; Day 21: –2.19). The results for the individual domains were consistent with those for the total score (Table S3 in Additional file 2).

### Sustained symptom alleviation

**Fig. 2:**
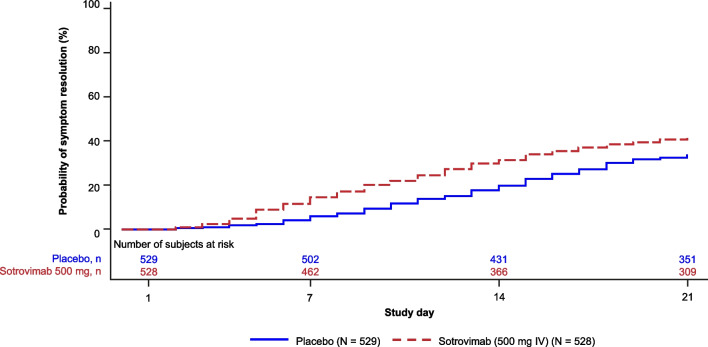
Time to sustained (≥ 48 h) symptom alleviation through Day 21. Participants who never achieve sustained (≥ 48 h) symptom alleviation (two or more, non-missing, consecutive scored questionnaires that showed symptom alleviation) are censored at day of withdrawal or Day 21, whichever was earliest. *IV* intravenous

**Fig. 3:**
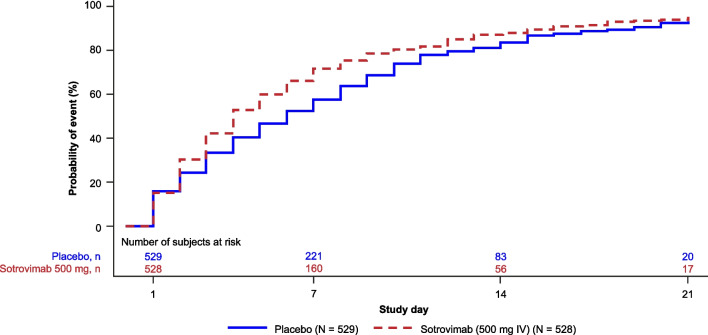
Post-hoc analysis: time to symptom alleviation (FLU-PRO user manual definition). Based on data through Day 21, as shown in the end of study Week 24 database. *FLU-PRO* inFLUenza Patient-Reported Outcome, *IV* intravenous

The proportion of patients reaching sustained (≥ 48 h) symptom alleviation by Day 21 was higher in the sotrovimab arm (41%) than the placebo arm (34%; adjusted relative risk ratio, 1.21 [95% CI 1.03–1.41]) (Table 2). The probability of reaching sustained (≥ 48 h) symptom alleviation by Day 21 was statistically significantly higher in the sotrovimab arm than the placebo arm (log-rank test *p* value = 0.002) (Fig. 2, Table S4 in Additional file 2). It was not possible to calculate the median time to sustained symptom alleviation for either treatment arm due to the high proportion of missing data. In the post-hoc analysis, median time to symptom alleviation according to the FLU-PRO user manual definition was 6 days on placebo versus 4 days on sotrovimab (log-rank test *p* value < 0.001) (Fig. 3).

**Table 2:** Summary and analysis of the proportion of patients achieving sustained (≥ 48 h) symptom alleviation

	All patients		< 2 Moderate/higher symptom severity at baseline		≥ 2 Moderate/higher symptom severity at baseline	
	Placebo (N = 529)	Sotrovimab (N = 528)	Placebo (N = 237)	Sotrovimab (N = 241)	Placebo (N = 214)	Sotrovimab (N = 218)
*Day 7*						
Symptom alleviation status, n (%)						
Sustained (≥ 48 h) symptom alleviation	31 (6)	76 (14)	25 (11)	52 (22)	7 (3)	22 (10)
Not sustained symptom alleviation	498 (94)	452 (86)	212 (89)	189 (78)	207 (97)	196 (90)
Adjusted relative risk ratio (95% CI)	2.51 (1.69 to 3.72)		2.07 (1.33 to 3.21)		3.10 (1.36 to 7.07)	
*p* value	< 0.001		0.002		0.008	
Adjusted relative risk difference	9.99		13.46		8.72	
*Day 14*						
Symptom alleviation status, n (%)						
Sustained (≥ 48 h) symptom alleviation	104 (20)	164 (31)	62 (26)	96 (40)	36 (17)	63 (29)
Not sustained symptom alleviation	425 (80)	364 (69)	175 (74)	145 (60)	178 (83)	155 (71)
Adjusted relative risk ratio (95% CI)	1.59 (1.28 to 1.97)		1.52 (1.17 to 1.98)		1.72 (1.20 to 2.47)	
*p* value	< 0.001		0.002		0.004	
Adjusted relative risk difference	8.08		11.20		10.06	
*Day 21*						
Symptom alleviation status, n (%)						
Sustained (≥ 48 h) symptom alleviation	178 (34)	214 (41)	104 (44)	126 (52)	64 (30)	78 (36)
Not sustained symptom alleviation	351 (66)	314 (59)	133 (56)	115 (48)	150 (70)	140 (64)
Adjusted relative risk ratio (95% CI)	1.21 (1.03 to 1.41)		1.19 (0.99 to 1.43)		1.20 (0.92 to 1.57)	
*p* value	0.020		0.072		0.188	
Adjusted relative risk difference	4.37		6.31		4.61	

The analysis by symptom severity subgroup is based on data collected through Day 21, as shown in the end of study Week 24 database

Data where symptom alleviation cannot be assessed due to a missing/incomplete questionnaire are imputed as no symptom alleviation

Analysis was performed using a Poisson model, adjusting for region (Europe, North America, South America), duration of symptoms (≤ 3 days versus ≥ 4 days), age (≤ 70 versus > 70 years), and sex (male, female). For the analysis by symptom severity subgroup, a treatment by patient-reported symptom severity interaction term was added to the above model

*CI* confidence interval

The estimates observed in the subgroup analyses were generally consistent with those for the overall population. In general, participants with ≥ 2 symptoms of moderate/higher intensity had a lower probability of achieving sustained (≥ 48 h) symptom alleviation than those with < 2 symptoms of moderate/higher intensity (Fig. S9 in Additional file 2). A higher proportion within each subgroup achieved sustained (≥ 48 h) symptom alleviation in the sotrovimab versus placebo arm at Day 7, 14, and 21 (Table 2).

### Other PRO measurements

All domain scores of the WPAI decreased over time in both arms (Fig. S10 in Additional file 2). There was little difference observed between the arms in any domain, and the small sample size (due to the low number of participants employed and number of questionnaires completed) made it difficult to draw definitive conclusions.

There was also little difference observed between the treatment arms in any of the eight domains of the SF-12, plus the Physical and Mental Component Summary score (Fig. S11 in Additional file 2). As for the WPAI, the small sample size precluded any definitive conclusions.

## Discussion

A previous report from the COMET-ICE study has demonstrated the efficacy of sotrovimab for improving rates of hospitalization and survival in high-risk COVID-19 patients [6]. Here, we show further evidence of the benefit of early treatment with sotrovimab in providing significant and more rapid improvement in patient-reported symptoms.

Based on AUC, sotrovimab provides statistically significant improvements through Day 21 in patient-reported symptoms compared with placebo. Based on the observed mean daily FLU-PRO Plus total and individual domain scores, improvements with sotrovimab occurred rapidly (within a week of treatment administration) compared to placebo, and the observed mean daily score for the individual domains of the FLU-PRO Plus followed a similar pattern. The clinical relevance of the PRO findings was further supported by estimates towards shorter time to symptom resolution in the sotrovimab arm compared to placebo. As COVID-19 vaccination and prior infection become increasingly widespread, further improvements in time to symptom resolution may occur. As seen previously in influenza, duration of illness is shortened among immune participants [18].

Sub-group analyses were generally consistent with results for the overall population, although interpretation of the data for some categories is limited due to the small numbers of participants completing the questionnaires. Results obtained with other PRO tools were less conclusive. Minimal difference between the sotrovimab and placebo arms was observed for any of the WPAI domains, and little difference was observed for any of the SF-12 domains or summary scores. It should be noted that these instruments are generic, and therefore may be less sensitive to change overall in a specific disease.

Although the COMET-ICE trial, as well as analyses of the content validity and psychometric properties of the FLU-PRO Plus [13], were conducted during a period when circulating variants caused more severe symptoms, the FLU-PRO Plus is appropriate for capturing milder symptoms with a shorter duration, as seen with current COVID-19 variants. The FLU-PRO Plus includes a broad spectrum of 34 symptoms in seven domains, with five response options that capture mild to severe symptoms.

The main limitation of the current analysis is the potential for bias due to missing questionnaire data. This was primarily due to operational challenges surrounding rapid initiation of the study which resulted from urgent medical need during the early stages of the pandemic. This led to a lack of time to sufficiently execute electronic PRO set-up and implement a back-up paper plan. However, despite these challenges, completion rates were similar between both treatment arms, with more than 80% of participants completing the questionnaire on Day 1 and approximately 50% at Day 21. In addition, no single minimal clinically important difference for FLU-PRO in COVID-19 has been established, and was not developed as part of this study.

## Conclusions

The COMET-ICE trial was designed to test pandemic-relevant serious clinical outcomes (hospitalization or death) and demonstrated a clinical benefit of sotrovimab treatment. The current study adds to these findings by demonstrating significant and rapid improvements in patient-reported COVID-19 symptoms, as measured by the FLU-PRO Plus questionnaire in both total and individual domain scores, with patients noticing a benefit within a week of treatment administration.

### Supplementary Information

**Additional file 1**. CONSORT Checklist.

**Additional file 2**. Supplementary Data.

## Data Availability

The datasets generated and/or analyzed during the current study are not publicly available but are available from the corresponding author on reasonable request.

## Abbreviations

AUC: Area under the curve
BMI: Body mass index
CI: Confidence interval
COMET-ICE: **CO**vid-19 **M**onoclonal antibody **E**fficacy **T**rial-**I**ntent to **C**are **E**arly
FDA: U.S. Food and Drug Administration
FLU-PRO (Plus): InFLUenza Patient-Reported Outcome (Plus)
PRO: Patient-reported outcome
RT-PCR: Reverse transcription polymerase chain reaction
SARS: Severe acute respiratory syndrome
SF-12: Short Form-12
WPAI: Work Productivity and Activity Impairment
