# Supplementary material for: Assessment of symptoms in COMET-ICE, a phase 2/3 study of sotrovimab for early treatment of non-hospitalized patients with COVID-19
Source: J Patient Rep Outcomes. 2023 Sep 13;7:92. doi: 10.1186/s41687-023-00621-8 (PMC10499766; doi:10.1186/s41687-023-00621-8)
Supplement: Supplementary file 2 — Additional file 2. Supplementary Data. [file 41687_2023_621_MOESM2_ESM.docx]

# Supporting information: Additional file 1

**Assessment of symptoms in COMET-ICE, a phase 2/3 study of sotrovimab for early treatment of non-hospitalized patients with COVID-19**

**Table of Contents**

Definition of sustained symptom alleviation (i.e., responder definition) 2

Table S1 FLU-PRO Plus questionnaire availability and format 4

Table S2 Change from baseline of COVID-19-related illness as measured by FLU-PRO Plus (total and domain scores) for overall population 5

Table S3 Average change from baseline (AUC) of COVID-19-related illness as measured by FLU-PRO Plus (total and domain scores) by symptom severity subgroup 8

Table S4 Summary and analysis of time to sustained (≥ 48 hours) symptom alleviation 12

Fig. S1 Secondary endpoints testing hierarchy 13

Fig. S2 Observed mean daily FLU-PRO Plus “Nose” domain score by study day 14

Fig. S3 Observed mean daily FLU-PRO Plus “Throat” domain score by study day 15

Fig. S4 Observed mean daily FLU-PRO Plus “Eyes” domain score by study day 16

Fig. S5 Observed mean daily FLU-PRO Plus “Chest/Respiratory” domain score by study day 17

Fig. S6 Observed mean daily FLU-PRO Plus “Gastrointestinal” domain score by study day 18

Fig. S7 Observed mean daily FLU-PRO Plus “Body/Systemic” domain score by study day 19

Fig. S8 Observed mean daily FLU-PRO Plus “Sense” domain score by study day 20

Fig. S9 Time to sustained (≥ 48 h) symptom alleviation through Day 21, by symptom severity subgroup 21

Fig. S10 Mean (+/- SD) change from baseline in WPAI scores 22

Fig. S11 Mean (+/- SD) change from baseline in SF-12 Hybrid domain scores 23

#### Definition of sustained symptom alleviation (i.e., responder definition)

In September 2020, FDA released Guidance (https://www.fda.gov/media/142143/download) encouraging the development and use by study sponsors of a responder definition. To develop this responder definition FLU-PRO Plus items have been mapped onto the item suggested by the FDA as follows:

| Item number | FDA COVID-19 example items | FDA example response options | FLU-PRO Plus questions (matching) | FLU-PRO Plus response (matching) | FLU-PRO Plus response options |
| --- | --- | --- | --- | --- | --- |
| 1 | Stuffy or runny nose | None = 0 Mild = 1 Moderate = 2 Severe = 3 | Congested or stuffy nose |  | Not at all A little bit Somewhat Quite a bit Very much |
| 2 | Sore throat |  | Sore or painful throat |  |  |
| 3 | Shortness of breath |  | Trouble breathing |  |  |
| 4 | Cough (severity) |  | Dry or hacking cough (severity) | Wet or loose cough (severity) |  |
| 5 | Low energy or tiredness |  | Weak or tired |  |  |
| 6 | Muscle or body aches |  | Body aches or pains |  |  |
| 7 | Headache |  | Headache |  |  |
| 8 | Chills or shivering |  | Chills or shivering |  |  |
| 9 | Feeling hot or feverish |  | Felt hot |  |  |
| 10 | Nausea (feeling like you want to throw up) |  | Felt nauseous (feeling like you wanted to throw up) |  |  |
| 11 | How many times did you vomit (throw up) in the last 24 hours? | I did not vomit at all = 0 1–2 times = 1 3–4 times = 2 5 or more times = 3 | How many times did you vomit? |  | 0 times  1 time  2 times  3 times  4 or more times |
| 12 | How many times did you have diarrhea (loose or watery stools) in the last 24 hours? | I did not have diarrhea at all = 0 1–2 times = 1 3–4 times = 2 5 or more times = 3 | How many times did you have diarrhea? |  | 0 times  1 time  2 times  3 times  4 or more times |
| 13 | Rate your sense of smell in the last 24 hours | My sense of smell is THE SAME as usual  = 0 My sense of smell is LESS than usual = 1 I have NO sense of smell = 2 | Loss of smell |  | Yes No |
| 14 | Rate your sense of taste in the last 24 hours | My sense of taste is THE SAME as usual = 0 My sense of taste is LESS than usual = 1 I have NO sense of taste = 2 | Loss of taste |  |  |

*FDA*, U.S. Food and Drug Administration, *FLU-PRO Plus*, inFLUenza Patient-Reported Outcome Plus

A responder is defined as:

- Items 1–3 and 6–10 scoring “not at all”
- Items 4–5 (cough and fatigue) scoring no more than “somewhat” in severity
- Items 11–12 (vomiting and diarrhea) scoring “0 times”
- Items 13–14 (loss of smell or taste) scoring “yes” or “no”.

#### Table S1 FLU-PRO Plus questionnaire availability and format

|  | **Placebo (N = 529)** | **Sotrovimab  (N = 528)** |
| --- | --- | --- |
| *Day 7, N* | 525 | 522 |
| Available questionnaires, n (%) | 292 (56) | 296 (57) |
| ePRO | 256 (49) | 262 (50) |
| Paper/mixture | 36 (7) | 34 (7) |
| Incomplete questionnaires | 2 (< 1) | 2 (< 1) |
| ePRO | 0 | 0 |
| Paper/mixture | 2 (< 1) | 2 (< 1) |
| Missing questionnaires | 213 (44) | 224 (43) |
| *Day 14, N* | 524 | 522 |
| Available questionnaires, n (%) | 288 (55) | 265 (51) |
| ePRO | 254 (48) | 236 (45) |
| Paper/mixture | 34 (6) | 29 (6) |
| Incomplete questionnaires | 0 | 0 |
| ePRO | 0 | 0 |
| Paper/mixture | 0 | 0 |
| Missing questionnaires | 236 (45) | 257 (49) |
| *Day 21, N* | 520 | 521 |
| Available questionnaires, n (%) | 250 (48) | 262 (50) |
| ePRO | 222 (43) | 245 (47) |
| Paper/mixture | 28 (5) | 17 (3) |
| Incomplete questionnaires | 1 (< 1) | 0 |
| ePRO | 0 | 0 |
| Paper/mixture | 1 (<1) | 0 |
| Missing questionnaires | 269 (52) | 259 (50) |

*ePRO* electronic patient-reported outcome, *FLU-PRO Plus* inFLUenza Patient-Reported Outcome Plus

#### Table S2 Change from baseline of COVID-19-related illness as measured by FLU-PRO Plus (total and domain scores) for overall population

|  | | **Overall population** | |
| --- | --- | --- | --- |
|  |  | **Placebo**  **(N = 529)** | **Sotrovimab (500 mg IV) (N = 528)** |
| *Total* | | | |
| Change from baseline to  Day 7 | n | 399 | 412 |
|  | Mean (95% CI) | –0.51 (–0.56 to –0.46) | –0.73 (–0.78 to –0.68) |
|  | Difference (95% CI) | –0.22 (–0.29 to –0.15) | |
|  | *p* value | < 0.001 | |
| Change from baseline to  Day 14 | n | 373 | 385 |
|  | Mean (95% CI) | –0.84 (–0.88 to –0.79) | –0.95 (–0.99 to –0.91) |
|  | Difference (95% CI) | –0.11 (–0.17 to –0.05) | |
|  | *p* value | < 0.001 | |
| Change from baseline to  Day 21 | n | 345 | 379 |
|  | Mean (95% CI) | –0.96 (–1.00 to –0.92) | –1.03 (–1.07 to –0.99) |
|  | Difference (95% CI) | –0.07 (–0.13 to –0.02) | |
|  | *p* value | 0.010 | |
| *Nose* | | | |
| Change from baseline to  Day 7 | n | 401 | 412 |
|  | Mean (95% CI) | –0.59 (–0.66 to –0.53) | –0.77 (–0.84 to –0.71) |
|  | Difference (95% CI) | –0.18 (–0.27 to –0.09) | |
|  | *p* value | < 0.001 | |
| Change from baseline to  Day 14 | n | 375 | 385 |
|  | Mean (95% CI) | –0.94 (–0.99 to –0.88) | –1.00 (–1.05 to –0.94) |
|  | Difference (95% CI) | –0.06 (–0.14 to 0.02) | |
|  | *p* value | 0.124 | |
| Change from baseline to  Day 21 | n | 347 | 379 |
|  | Mean (95% CI) | –1.04 (–1.09 to –0.99) | –1.08 (–1.13 to –1.03) |
|  | Difference (95% CI) | –0.04 (–0.11 to 0.03) | |
|  | *p* value | 0.222 | |
| *Throat* | | | |
| Change from baseline to  Day 7 | n | 401 | 412 |
|  | Mean (95% CI) | –0.56 (–0.62 to –0.50) | –0.68 (–0.74 to –0.62) |
|  | Difference (95% CI) | –0.12 (–0.21 to –0.04) | |
|  | *p* value | 0.005 | |
| Change from baseline to  Day 14 | n | 375 | 385 |
|  | Mean (95% CI) | –0.78 (–0.83 to –0.74) | –0.89 (–0.94 to –0.84) |
|  | Difference (95% CI) | –0.10 (–0.17 to –0.04) | |
|  | *p* value | 0.002 | |
| Change from baseline to  Day 21 | n | 347 | 379 |
|  | Mean (95% CI) | –0.86 (–0.90 to –0.81) | –0.92 (–0.97 to –0.88) |
|  | Difference (95% CI) | –0.07 (–0.13 to 0.00) | |
|  | *p* value | 0.042 | |
| *Eyes* | | | |
| Change from baseline to  Day 7 | n | 401 | 412 |
|  | Mean (95% CI) | –0.41 (–0.47 to –0.35) | –0.56 (–0.62 to –0.50) |
|  | Difference (95% CI) | –0.15 (–0.23 to –0.07) | |
|  | *p* value | < 0.001 | |
| Change from baseline to  Day 14 | n | 375 | 385 |
|  | Mean (95% CI) | –0.63 (–0.67 to –0.58) | –0.68 (–0.73 to –0.64) |
|  | Difference (95% CI) | –0.06 (–0.12 to 0.01) | |
|  | *p* value | 0.094 | |
| Change from baseline to  Day 21 | n | 347 | 379 |
|  | Mean (95% CI) | –0.68 (–0.73 to –0.64) | –0.74 (–0.79 to –0.70) |
|  | Difference (95% CI) | –0.06 (–0.12 to 0.01) | |
|  | *p* value | 0.085 | |
| *Chest/respiratory* | | | |
| Change from baseline to  Day 7 | n | 401 | 412 |
|  | Mean (95% CI) | –0.37 (–0.43 to –0.31) | –0.65 (–0.71 to –0.59) |
|  | Difference (95% CI) | –0.28 (–0.37 to –0.20) | |
|  | *p* value | < 0.001 | |
| Change from baseline to  Day 14 | n | 375 | 385 |
|  | Mean (95% CI) | –0.72 (–0.77 to –0.66) | –0.88 (–0.93 to –0.82) |
|  | Difference (95% CI) | –0.16 (–0.24 to –0.08) | |
|  | *p* value | < 0.001 | |
| Change from baseline to  Day 21 | n | 347 | 379 |
|  | Mean (95% CI) | –0.86 (–0.92 to –0.81) | –1.00 (–1.05 to –0.95) |
|  | Difference (95% CI) | –0.14 (–0.21 to –0.06) | |
|  | *p* value | < 0.001 | |
| *Gastrointestinal* | | | |
| Change from baseline to  Day 7 | n | 399 | 412 |
|  | Mean (95% CI) | –0.36 (–0.41 to –0.31) | –0.50 (–0.55 to –0.45) |
|  | Difference (95% CI) | –0.14 (–0.21 to –0.07) | |
|  | *p* value | < 0.001 | |
| Change from baseline to  Day 14 | n | 373 | 385 |
|  | Mean (95% CI) | –0.57 (–0.61 to –0.54) | –0.64 (–0.67 to –0.60) |
|  | Difference (95% CI) | –0.06 (–0.11 to –0.01) | |
|  | *p* value | < 0.014 | |
| Change from baseline to  Day 21 | n | 345 | 379 |
|  | Mean (95% CI) | –0.67 (–0.70 to –0.64) | –0.69 (–0.71 to –0.66) |
|  | Difference (95% CI) | –0.02 (–0.06 to 0.02) | |
|  | *p* value | 0.383 | |
| *Body/systemic* | | | |
| Change from baseline to  Day 7 | n | 400 | 412 |
|  | Mean (95% CI) | –0.64 (–0.70 to –0.58) | –0.91 (–0.98 to –0.85) |
|  | Difference (95% CI) | –0.28 (–0.36 to –0.19) | |
|  | *p* value | < 0.001 | |
| Change from baseline to  Day 14 | n | 374 | 385 |
|  | Mean (95% CI) | –1.04 (–1.09 to –0.99) | –1.17 (–1.22 to –1.12) |
|  | Difference (95% CI) | –0.13 (–0.20 to –0.06) | |
|  | *p* value | < 0.001 | |
| Change from baseline to  Day 21 | n | 346 | 379 |
|  | Mean (95% CI) | –1.19 (–1.24 to –1.15) | –1.26 (–1.30 to –1.22) |
|  | Difference (95% CI) | –0.07 (–0.13 to –0.01) | |
|  | *p* value | 0.031 | |
| *Sense* | | | |
| Change from baseline to  Day 7 | n | 399 | 412 |
|  | Mean (95% CI) | –0.07 (–0.11 to –0.03) | –0.16 (–0.20 to –0.12) |
|  | Difference (95% CI) | –0.10 (–0.15 to –0.04) | |
|  | *p* value | < 0.001 | |
| Change from baseline to  Day 14 | n | 373 | 385 |
|  | Mean (95% CI) | –0.22 (–0.27 to –0.18) | –0.31 (–0.35 to –0.27) |
|  | Difference (95% CI) | –0.08 (–0.14 to –0.02) | |
|  | *p* value | 0.005 | |
| Change from baseline to  Day 21 | n | 345 | 379 |
|  | Mean (95% CI) | –0.31 (–0.35 to –0.27) | –0.36 (–0.40 to –0.32) |
|  | Difference (95% CI) | –0.05 (–0.10 to 0.00) | |
|  | *p* value | 0.072 | |

Total score analysis through Day 7 is a secondary endpoint, part of the testing hierarchy. Analysis through Day 14 and 21 (total and domain scores) are exploratory endpoints. Analysis was performed using an ANCOVA model, adjusting for region (Europe, North America, South America), duration of symptoms (≤ 3 days versus ≥ 4 days), age (≤ 70 versus > 70 years), sex (male, female), and baseline score

*ANCOVA* analysis of covariance, *CI* confidence interval, *FLU-PRO Plus* inFLUenza Patient-Reported Outcome Plus, *IV* intravenous

#### Table S3 Average change from baseline (AUC) of COVID-19-related illness as measured by FLU-PRO Plus (total and domain scores) by symptom severity subgroup

|  | | **< 2 Moderate/higher symptom severity at baseline** | | **≥ 2 Moderate/higher symptom severity at baseline** | |
| --- | --- | --- | --- | --- | --- |
|  |  | **Placebo**  **(N = 237)** | **Sotrovimab (500 mg IV) (N = 241)** | **Placebo**  **(N = 214)** | **Sotrovimab (500 mg IV) (N = 218)** |
| *Total* | | | | | |
| AUC to Day 7 | n | 214 | 225 | 201 | 204 |
|  | Mean (95% CI) | –1.89 (–2.22 to –1.57) | –3.11 (–3.43 to –2.79) | –2.08 (–2.41 to –1.74) | –2.81 (–3.15 to –2.48) |
|  | Difference (95% CI) | –1.22 (–1.64 to –0.79) | | –0.74 (–1.18 to –0.29) | |
|  | *p* value | < 0.001 | | 0.002 | |
| AUC to Day 14 | n | 204 | 213 | 185 | 188 |
|  | Mean (95% CI) | –6.83 (–7.50 to –6.15) | –9.42 (–10.08 to –8.75) | –7.23 (–7.94 to –6.51) | –8.87 (–9.58 to –8.16) |
|  | Difference (95% CI) | –2.59 (–3.48 to –1.69) | | –1.65 (–2.59 to –0.70) | |
|  | *p* value | < 0.001 | | < 0.001 | |
| AUC to Day 21 | n | 185 | 209 | 174 | 187 |
|  | Mean (95% CI) | –13.01 (–14.03 to –12.00) | –16.30 (–17.27 to –15.33) | –13.56 (–14.62 to –12.51) | –15.75 (–16.78 to –14.73) |
|  | Difference (95% CI) | –3.29 (–4.62 to –1.96) | | –2.19 (–3.57 to –0.81) | |
|  | *p* value | < 0.001 | | 0.002 | |
| *Nose* | | | | | |
| AUC to Day 7 | n | 215 | 225 | 201 | 204 |
|  | Mean (95% CI) | –2.45 (–2.86 to –2.04) | –3.49 (–3.89 to –3.09) | –1.94 (–2.37 to –1.50) | –2.32 (–2.74 to –1.90) |
|  | Difference (95% CI) | –1.04 (–1.60 to –0.48) | | –0.38 (–0.97 to 0.20) | |
|  | *p* value | < 0.001 | | 0.201 | |
| AUC to Day 14 | n | 205 | 213 | 185 | 188 |
|  | Mean (95% CI) | –7.95 (–8.77 to –7.13) | –10.25 (–11.05 to –9.44) | –7.83 (–8.70 to –6.95) | –8.35 (–9.20 to –7.50) |
|  | Difference (95% CI) | –2.30 (–3.42 to –1.17) | | –0.52 (–1.71 to 0.68) | |
|  | *p* value | < 0.001 | | 0.394 | |
| AUC to Day 21 | n | 186 | 209 | 174 | 187 |
|  | Mean (95% CI) | –14.83 (–16.05 to –13.60) | –17.50 (–18.66 to –16.34) | –14.31 (–15.59 to –13.03) | –15.15 (–16.37 to –13.94) |
|  | Difference (95% CI) | –2.67 (–4.32 to –1.01) | | –0.84 (–2.58 to 0.89) | |
|  | *p* value | 0.002 | | 0.340 | |
| *Throat* | | | | | |
| AUC to Day 7 | n | 215 | 226 | 201 | 204 |
|  | Mean (95% CI) | –2.28 (–2.69 to –1.87) | –3.42 (–3.81 to –3.02) | –2.09 (–2.52 to –1.67) | –2.38 (–2.80 to –1.96) |
|  | Difference (95% CI) | –1.14 (–1.69 to –0.58) | | –0.29 (–0.87 to 0.30) | |
|  | *p* value | < 0.001 | | 0.336 | |
| AUC to Day 14 | n | 205 | 214 | 185 | 188 |
|  | Mean (95% CI) | –7.16 (–7.94 to –6.38) | –9.45 (–10.21 to –8.68) | –6.78 (–7.61 to –5.95) | –7.74 (–8.55 to –6.93) |
|  | Difference (95% CI) | –2.28 (–3.36 to –1.21) | | –0.96 (–2.11 to 0.18) | |
|  | *p* value | < 0.001 | | 0.100 | |
| AUC to Day 21 | n | 186 | 210 | 174 | 187 |
|  | Mean (95% CI) | –13.00 (–14.14 to –11.86) | –15.78 (–16.85 to –14.71) | –12.29 (–13.48 to –11.11) | –13.81 (–14.94 to –12.68) |
|  | Difference (95% CI) | –2.78 (–4.32 to –1.24) | | –1.51 (–3.13 to 0.10) | |
|  | *p* value | < 0.001 | | 0.066 | |
| *Eyes* | | | | | |
| AUC to Day 7 | n | 215 | 226 | 201 | 204 |
|  | Mean (95% CI) | –1.61 (–1.98 to –1.23) | –2.55 (–2.92 to –2.18) | –1.48 (–1.87 to –1.09) | –1.95 (–2.34 to –1.57) |
|  | Difference (95% CI) | –0.94 (–1.46 to –0.42) | | –0.47 (–1.01 to 0.07) | |
|  | *p* value | < 0.001 | | 0.088 | |
| AUC to Day 14 | n | 205 | 214 | 185 | 188 |
|  | Mean (95% CI) | –5.32 (–6.05 to –4.60) | –7.40 (–8.12 to –6.68) | –5.28 (–6.05 to –4.50) | –6.29 (–7.05 to –5.53) |
|  | Difference (95% CI) | –2.07 (–3.08 to –1.07) | | –1.01 (–2.08 to 0.05) | |
|  | *p* value | < 0.001 | | 0.062 | |
| AUC to Day 21 | n | 186 | 210 | 174 | 187 |
|  | Mean (95% CI) | –9.73 (–10.82 to –8.63) | –12.44 (–13.49 to –11.40) | –9.87 (–11.01 to –8.73) | –11.19 (–12.29 to –10.09) |
|  | Difference (95% CI) | –2.72 (–4.21 to –1.22) | | –1.32 (–2.87 to 0.23) | |
|  | *p* value | < 0.001 | | 0.097 | |
| *Chest/respiratory* | | | | | |
| AUC to Day 7 | n | 215 | 226 | 201 | 204 |
|  | Mean (95% CI) | –1.44 (–1.81 to –1.08) | –2.79 (–3.15 to –2.43) | –1.28 (–1.66 to –0.90) | –2.22 (–2.60 to –1.84) |
|  | Difference (95% CI) | –1.35 (–1.85 to –0.85) | | –0.93 (–1.46 to –0.41) | |
|  | *p* value | < 0.001 | | < 0.001 | |
| AUC to Day 14 | n | 205 | 214 | 185 | 188 |
|  | Mean (95% CI) | –5.60 (–6.41 to –4.80) | –8.80 (–9.58 to –8.01) | –5.37 (–6.21 to –4.52) | –7.61 (–8.46 to –6.76) |
|  | Difference (95% CI) | –3.19 (–4.29 to –2.10) | | –2.25 (–3.40 to –1.09) | |
|  | *p* value | < 0.001 | | < 0.001 | |
| AUC to Day 21 | n | 186 | 210 | 174 | 187 |
|  | Mean (95% CI) | –11.39 (–12.61 to –10.17) | –15.59 (–16.75 to –14.44) | –10.79 (–12.04 to –9.53) | –14.24 (–15.47 to –13.01) |
|  | Difference (95% CI) | –4.20 (–5.84 to –2.56) | | –3.45 (–5.17 to –1.74) | |
|  | *p* value | < 0.001 | | < 0.001 | |
| *Gastrointestinal* | | | | | |
| AUC to Day 7 | n | 214 | 226 | 201 | 204 |
|  | Mean (95% CI) | –1.51 (–1.81 to –1.21) | –2.40 (–2.69 to –2.11) | –1.24 (–1.55 to –0.93) | –1.72 (–2.03 to –1.41) |
|  | Difference (95% CI) | –0.89 (–1.30 to –0.48) | | –0.48 (–0.91 to –0.05) | |
|  | *p* value | < 0.001 | | 0.028 | |
| AUC to Day 14 | n | 204 | 214 | 185 | 188 |
|  | Mean (95% CI) | –4.92 (–5.48 to –4.36) | –6.81 (–7.35 to –6.26) | –4.42 (–5.00 to –3.83) | –5.77 (–6.36 to –5.18) |
|  | Difference (95% CI) | –1.88 (–2.65 to –1.12) | | –1.35 (–2.17 to –0.54) | |
|  | *p* value | < 0.001 | | 0.002 | |
| AUC to Day 21 | n | 185 | 210 | 174 | 187 |
|  | Mean (95% CI) | –9.19 (–9.99 to –8.38) | –11.46 (–12.22 to –10.71) | –8.75 (–9.58 to –7.93) | –9.94 (–10.74 to –9.13) |
|  | Difference (95% CI) | –2.28 (–3.36 to –1.20) | | –1.18 (–2.31 to –0.05) | |
|  | *p* value | < 0.001 | | 0.041 | |
| *Body/systemic* | | | | | |
| AUC to Day 7 | n | 215 | 226 | 201 | 204 |
|  | Mean (95% CI) | –2.48 (–2.90 to –2.05) | –3.91 (–4.33 to –3.50) | –2.60 (–3.04 to –2.16) | –3.70 (–4.14 to –3.25) |
|  | Difference (95% CI) | –1.44 (–1.98 to –0.89) | | –1.09 (–1.66 to –0.53) | |
|  | *p* value | < 0.001 | | < 0.001 | |
| AUC to Day 14 | n | 205 | 214 | 185 | 188 |
|  | Mean (95% CI) | –8.89 (–9.75 to –8.03) | –11.70 (–12.55 to –10.85) | –9.00 (–9.91 to –8.09) | –11.24 (–12.16 to –10.32) |
|  | Difference (95% CI) | –2.81 (–3.91 to –1.71) | | –2.24 (–3.41 to –1.08) | |
|  | *p* value | < 0.001 | | < 0.001 | |
| AUC to Day 21 | n | 186 | 210 | 174 | 187 |
|  | Mean (95% CI) | –16.66 (–17.93 to –15.39) | –20.35 (–21.57 to –19.13) | –16.89 (–18.21 to –15.57) | –19.62 (–20.93 to –18.32) |
|  | Difference (95% CI) | –3.69 (–5.30 to –2.08) | | –2.73 (–4.41 to –1.05) | |
|  | *p* value | < 0.001 | | 0.002 | |
| *Sense* | | | | | |
| AUC to Day 7 | n | 214 | 226 | 201 | 204 |
|  | Mean (95% CI) | –0.21 (–0.45 to 0.03) | –0.84 (–1.07 to –0.61) | –0.02 (–0.27 to 0.22) | –0.18 (–0.43 to 0.06) |
|  | Difference (95% CI) | –0.63 (–0.96 to –0.30) | | –0.16 (–0.50 to 0.18) | |
|  | *p* value | < 0.001 | | 0.364 | |
| AUC to Day 14 | n | 204 | 214 | 185 | 188 |
|  | Mean (95% CI) | –1.39 (–1.94 to –0.83) | –2.94 (–3.48 to –2.41) | –1.00 (–1.58 to –0.42) | –1.51 (–2.08 to –0.93) |
|  | Difference (95% CI) | –1.56 (–2.32 to –0.79) | | –0.51 (–1.32 to 0.30) | |
|  | *p* value | < 0.001 | | 0.220 | |
| AUC to Day 21 | n | 185 | 210 | 174 | 187 |
|  | Mean (95% CI) | –3.75 (–4.62 to –2.88) | –5.76 (–6.57 to –4.95) | –2.84 (–3.73 to –1.94) | –3.41 (–4.27 to –2.55) |
|  | Difference (95% CI) | –2.01 (–3.19 to –0.83) | | –0.58 (–1.81 to 0.66) | |
|  | *p* value | < 0.001 | | 0.360 | |

The analysis by symptom severity subgroup is based on data collected through Day 21, as shown in the end of study Week 24 database. Analysis was performed using an ANCOVA model, adjusting for region (Europe, North America, South America), duration of symptoms (≤ 3 days versus ≥ 4 days), age (≤ 70 versus > 70 years), gender (male, female), baseline score, patient-reported symptom severity (< 2 versus ≥ 2) and treatment by patient-reported symptom severity interaction

*ANCOVA* analysis of covariance, *AUC* area under the curve, *CI* confidence interval, *FLU-PRO Plus* inFLUenza Patient-Reported Outcome Plus, *IV* intravenous

#### Table S4 Summary and analysis of time to sustained (≥ 48 hours) symptom alleviation

|  | **All patients** | | **< 2 Moderate/higher symptom severity at baseline** | | **≥ 2 Moderate/higher symptom severity at baseline** | |
| --- | --- | --- | --- | --- | --- | --- |
|  | **Placebo**  **(N = 529)** | **Sotrovimab**  **(N = 528)** | **Placebo**  **(N = 237)** | **Sotrovimab**  **(N = 241)** | **Placebo**  **(N = 214)** | **Sotrovimab**  **(N = 218)** |
| *Day 7* | | | | | | |
| Number of participants with event, n (%) | 31 (6) | 76 (14) | 25 (11) | 52 (22) | 7 (3) | 22 (10) |
| Number of participants censored, n (%) | 4 (< 1) | 6 (1) | 1 (< 1) | 2 (< 1) | 1 (< 1) | 0 (0) |
| Number of participants without event,  n (%) | 494 (93) | 446 (84) | 211 (89) | 187 (78) | 206 (96) | 196 (90) |
| Probability of sustained symptom alleviation by Day 7, % (95% CI) | 5.9  (4.2 to 8.3) | 14.5  (11.8 to 17.9) | 10.6 (7.3 to 15.2) | 21.7 (17.0 to 27.5) | 3.3 (1.6 to 6.8) | 10.1 (6.8 to 14.9) |
| *Day 14* | | | | | | |
| Number of participants with event, n (%) | 104 (20) | 164 (31) | 62 (26) | 96 (40) | 36 (17) | 63 (29) |
| Number of participants censored, n (%) | 5 (< 1) | 6 (1) | 2 (< 1) | 2 (< 1) | 1 (< 1) | 0 (0) |
| Number of participants without event,  n (%) | 420 (79) | 358 (68) | 173 (73) | 143 (59) | 177 (83) | 155 (71) |
| Probability of sustained symptom alleviation by Day 14, % (95% CI) | 19.8  (16.7 to 23.5) | 31.4 (27.6 to 35.6) | 26.3 (21.1 to 32.4) | 40.1 (34.2 to 46.6) | 16.9 (12.5 to 22.6) | 28.9 (23.4 to 35.4) |
| *Day 21* | | | | | | |
| Number of participants with event, n (%) | 178 (34) | 214 (41) | 104 (44) | 126 (52) | 64 (30) | 78 (36) |
| Number of participants censored, n (%) | 351 (66) | 314 (59) | 133 (56) | 115 (48) | 150 (70) | 140 (64) |
| Number of participants without event,  n (%) | 0 (0) | 0 (0) | 0 (0) | 0 (0) | 0 (0) | 0 (0) |
| Probability of sustained symptom alleviation by Day 21, % (95% CI) | 34.0 (30.1 to 38.2) | 41.0 (36.9 to 45.4) | 44.4 (38.3 to 51.0) | 52.7 (46.5 to 59.1) | 30.0 (24.4 to 36.7) | 35.8 (29.8 to 42.6) |
| Log-rank *p* value | 0.002 | | 0.011 | | 0.107 | |

Analysis was performed using a log-rank test stratified by region (Europe, North America, South America), duration of symptoms (≤ 3 days versus ≥ 4 days), age (≤ 70 versus > 70 years) and gender (male, female). The analysis by symptom severity subgroup was based on data through Day 21, as shown in the end of study Week 24 database

*CI* confidence interval

#### Fig. S1 Secondary endpoints testing hierarchy


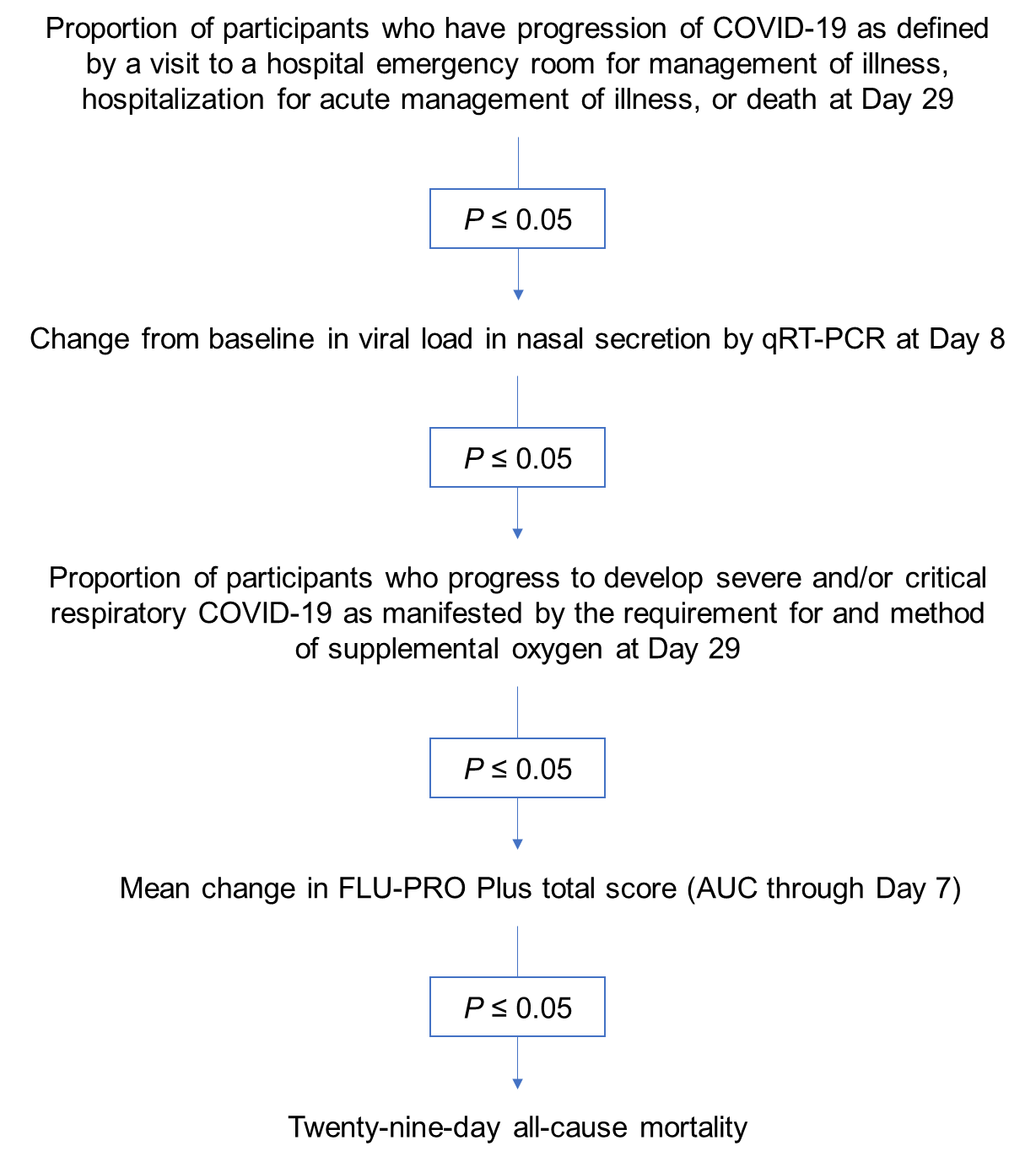


*AUC* area under the curve, *FLU-PRO Plus* inFLUenza Patient-Reported Outcome Plus, *qRT-PCR* quantitative real-time polymerase chain reaction

#### Fig. S2 Observed mean daily FLU-PRO Plus “Nose” domain score by study day


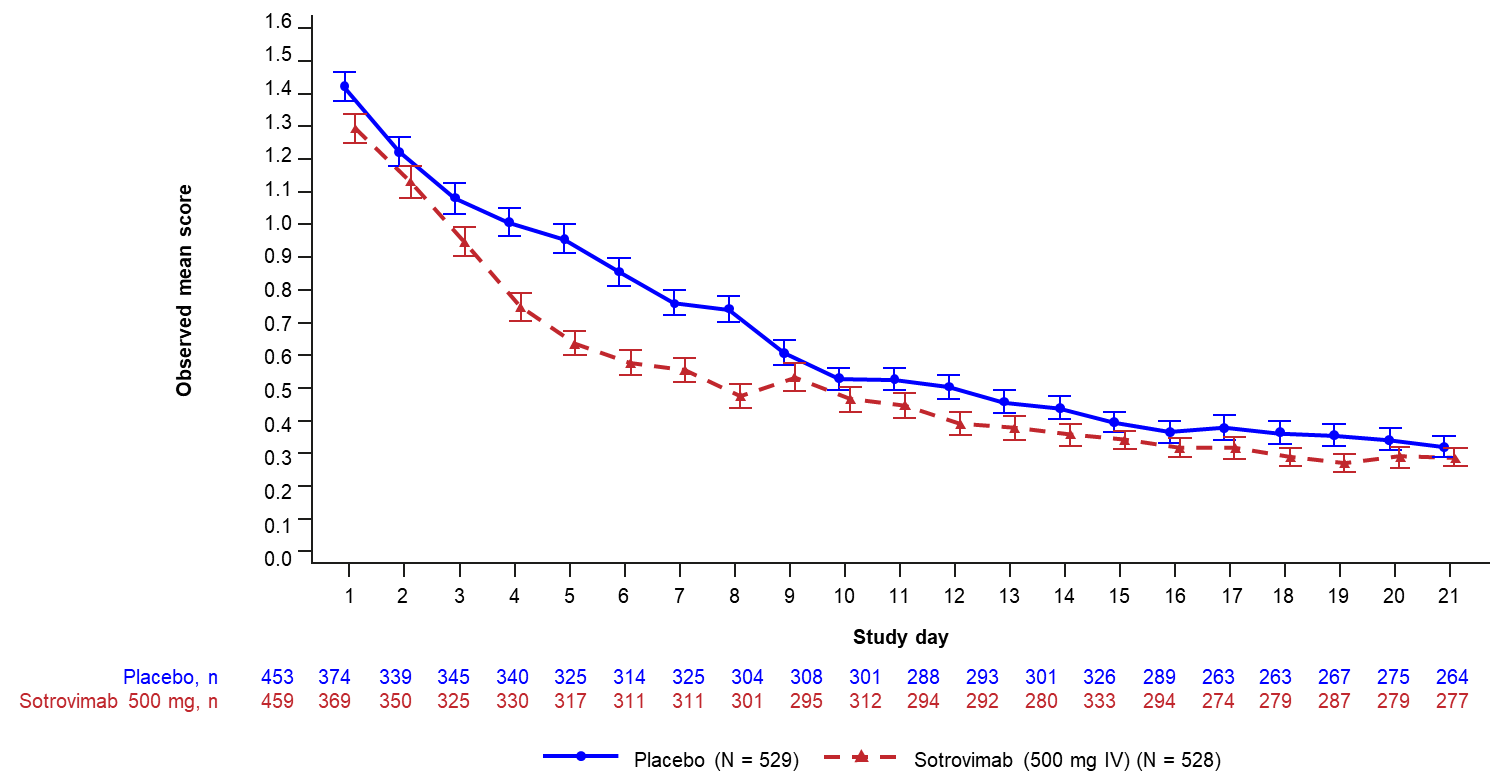


Based on data through Day 21, as shown in the end of study Week 24 database

*FLU-PRO Plus* inFLUenza Patient-Reported Outcome Plus, *IV* intravenous

#### Fig. S3 Observed mean daily FLU-PRO Plus “Throat” domain score by study day


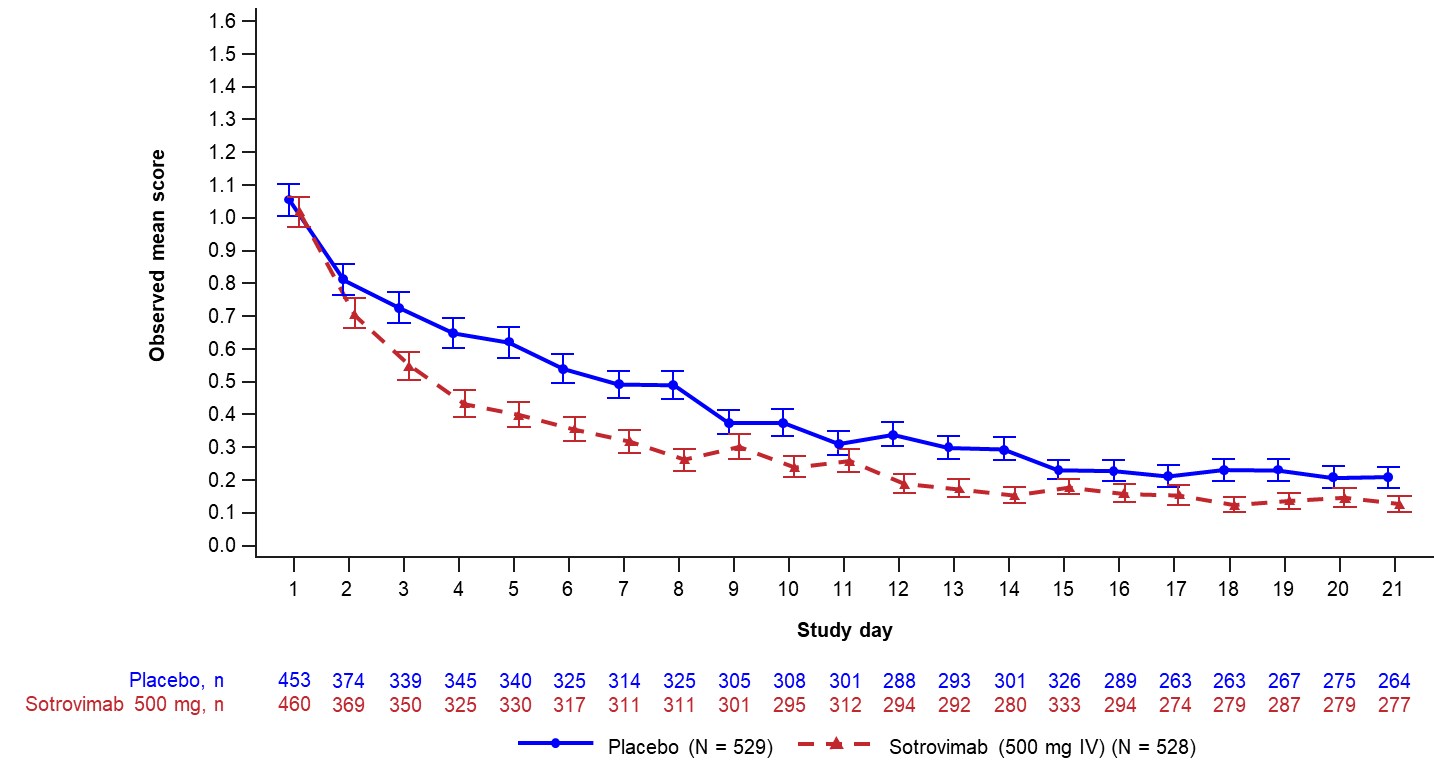


Based on data through Day 21, as shown in the end of study Week 24 database

*FLU-PRO Plus* inFLUenza Patient-Reported Outcome Plus, *IV* intravenous

#### Fig. S4 Observed mean daily FLU-PRO Plus “Eyes” domain score by study day


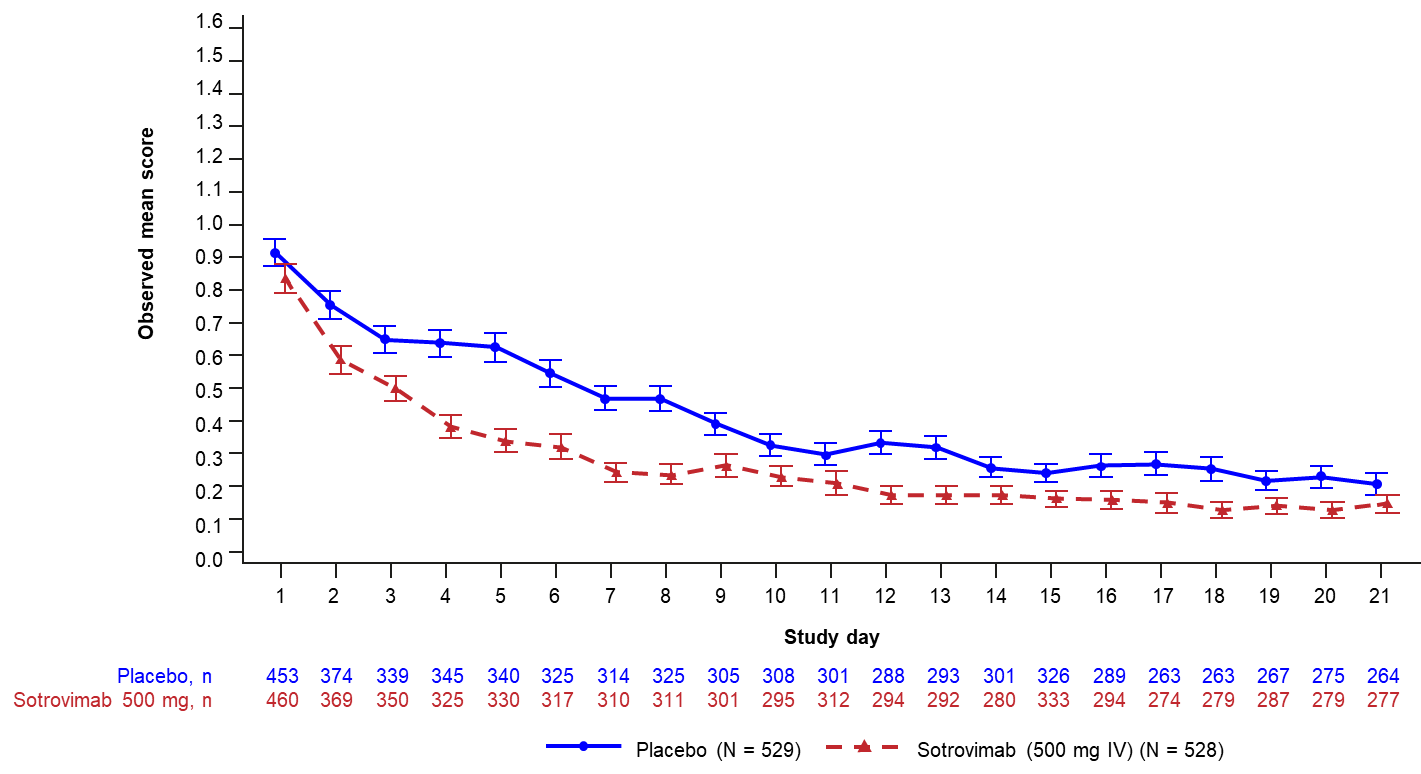


Based on data through Day 21, as shown in the end of study Week 24 database

*FLU-PRO Plus* inFLUenza Patient-Reported Outcome Plus, *IV* intravenous

#### Fig. S5 Observed mean daily FLU-PRO Plus “Chest/Respiratory” domain score by study day


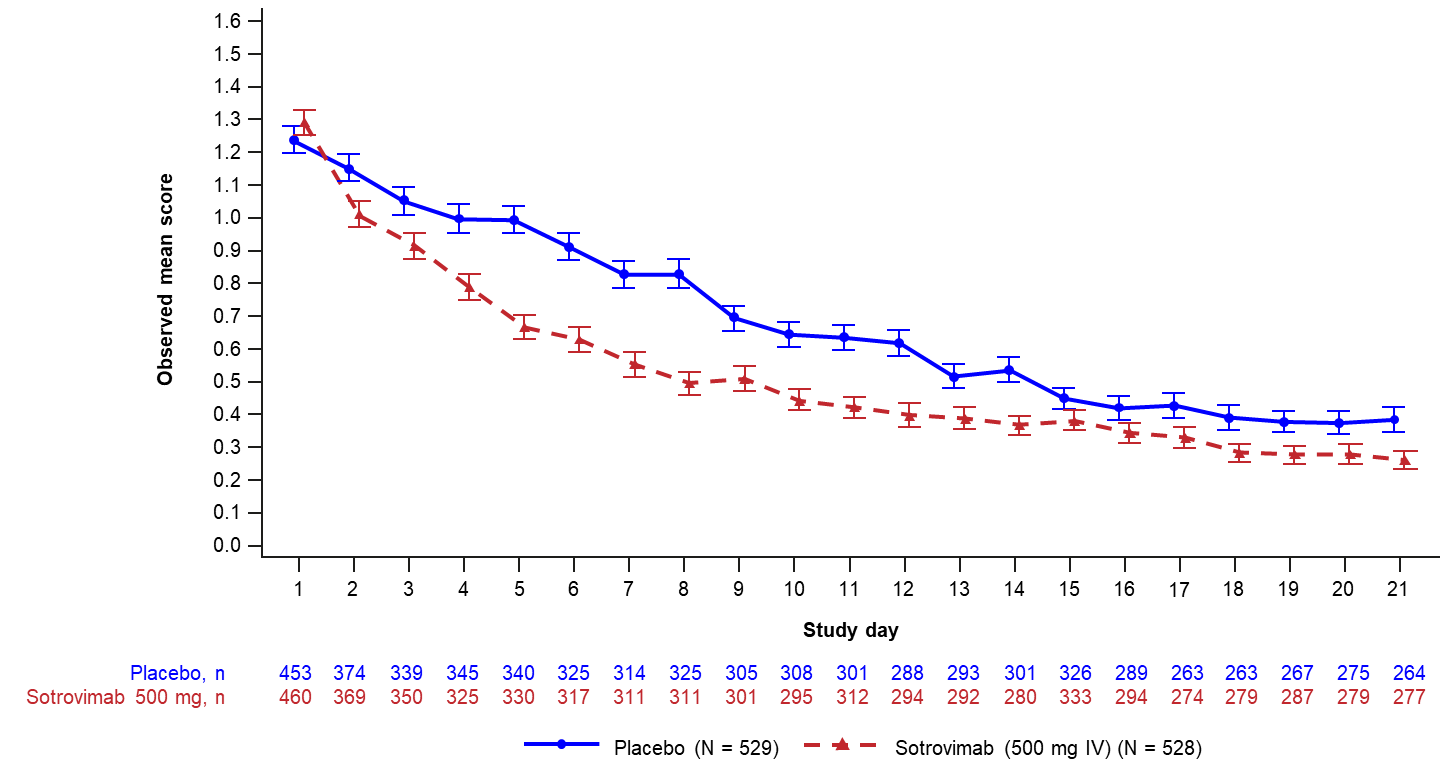


Based on data through Day 21, as shown in the end of study Week 24 database

*FLU-PRO Plus* inFLUenza Patient-Reported Outcome Plus, *IV* intravenous

#### Fig. S6 Observed mean daily FLU-PRO Plus “Gastrointestinal” domain score by study day


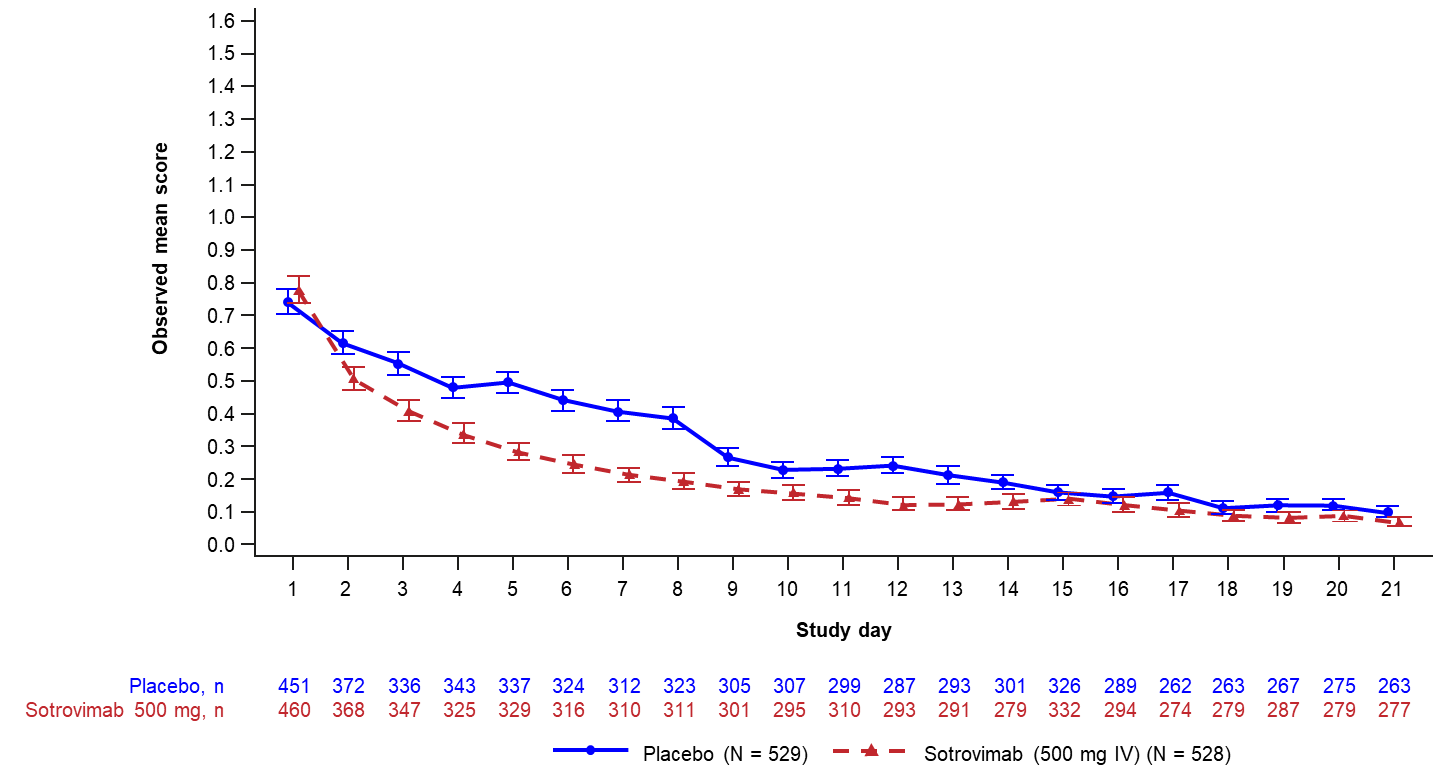


Based on data through Day 21, as shown in the end of study Week 24 database

*FLU-PRO Plus* inFLUenza Patient-Reported Outcome Plus, *IV* intravenous

#### Fig. S7 Observed mean daily FLU-PRO Plus “Body/Systemic” domain score by study day


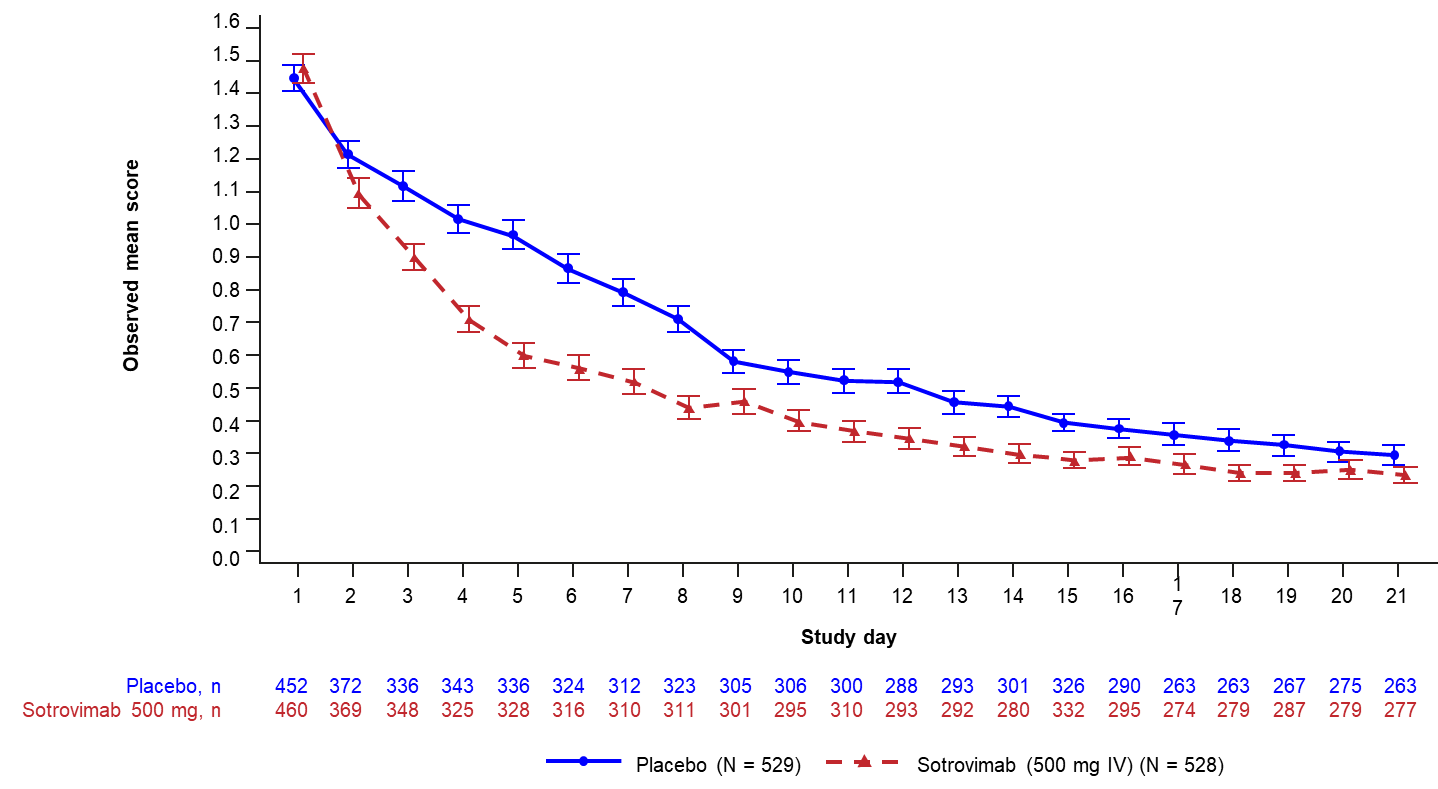


Based on data through Day 21, as shown in the end of study Week 24 database

*FLU-PRO Plus* inFLUenza Patient-Reported Outcome Plus, *IV* intravenous

#### Fig. S8 Observed mean daily FLU-PRO Plus “Sense” domain score by study day


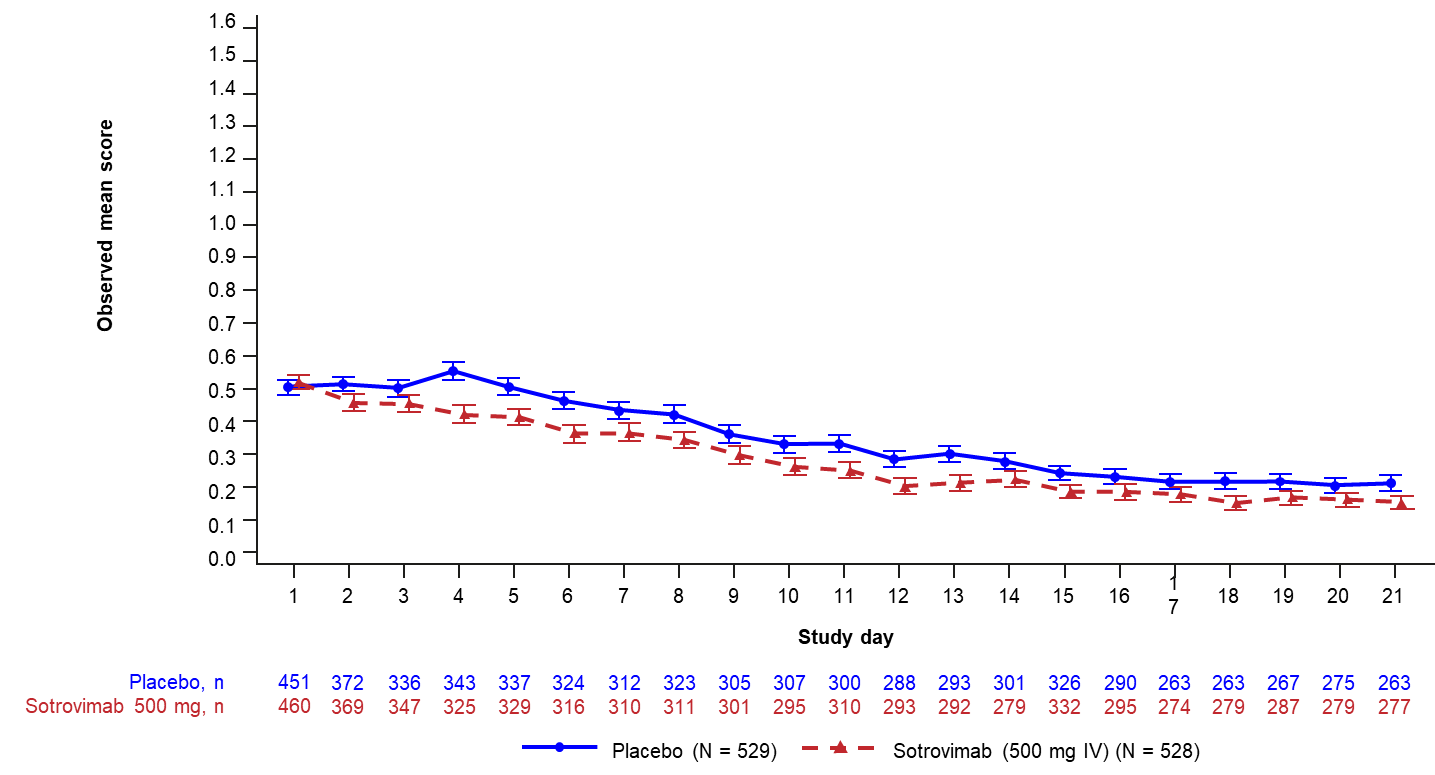


Based on data through Day 21, as shown in the end of study Week 24 database

*FLU-PRO Plus* inFLUenza Patient-Reported Outcome Plus, *IV* intravenous

#### Fig. S9 Time to sustained (≥ 48 h) symptom alleviation through Day 21, by symptom severity subgroup


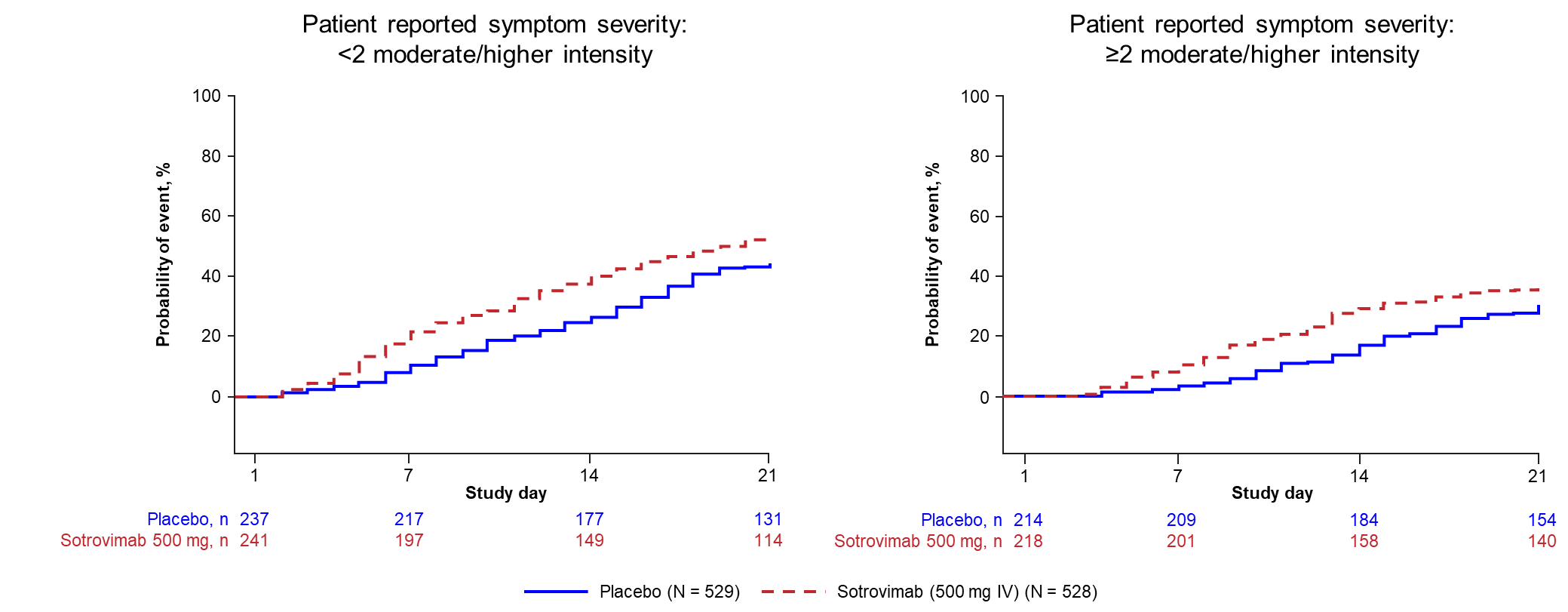


Based on data through Day 21, as shown in the end of study Week 24 database

*IV* intravenous

#### Fig. S10 Mean (+/- SD) change from baseline in WPAI scores


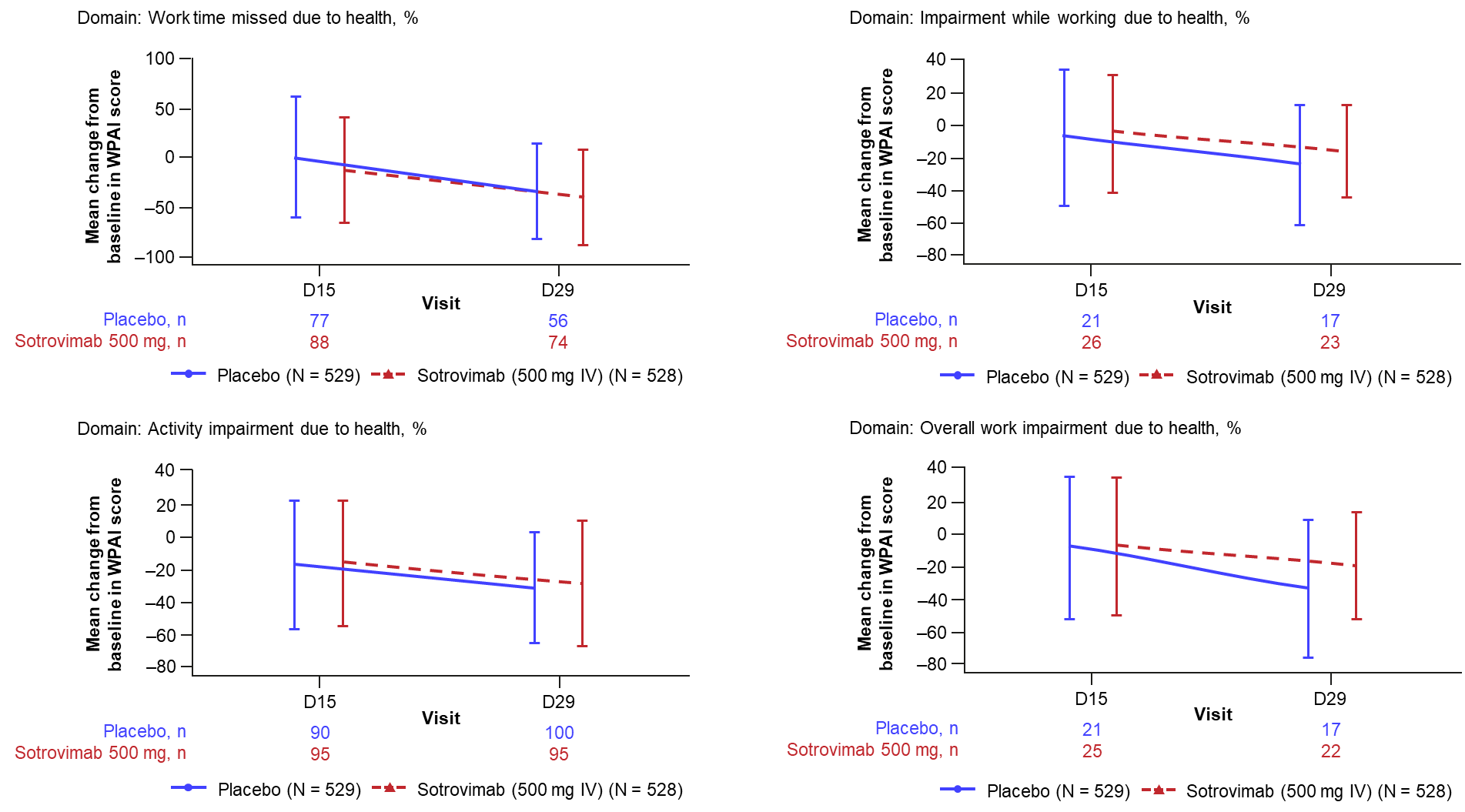


*D* day, *IV* intravenous, *SD* standard deviation, *WPAI* Work Productivity and Activity Impairment

#### Fig. S11 Mean (+/- SD) change from baseline in SF-12 Hybrid domain scores


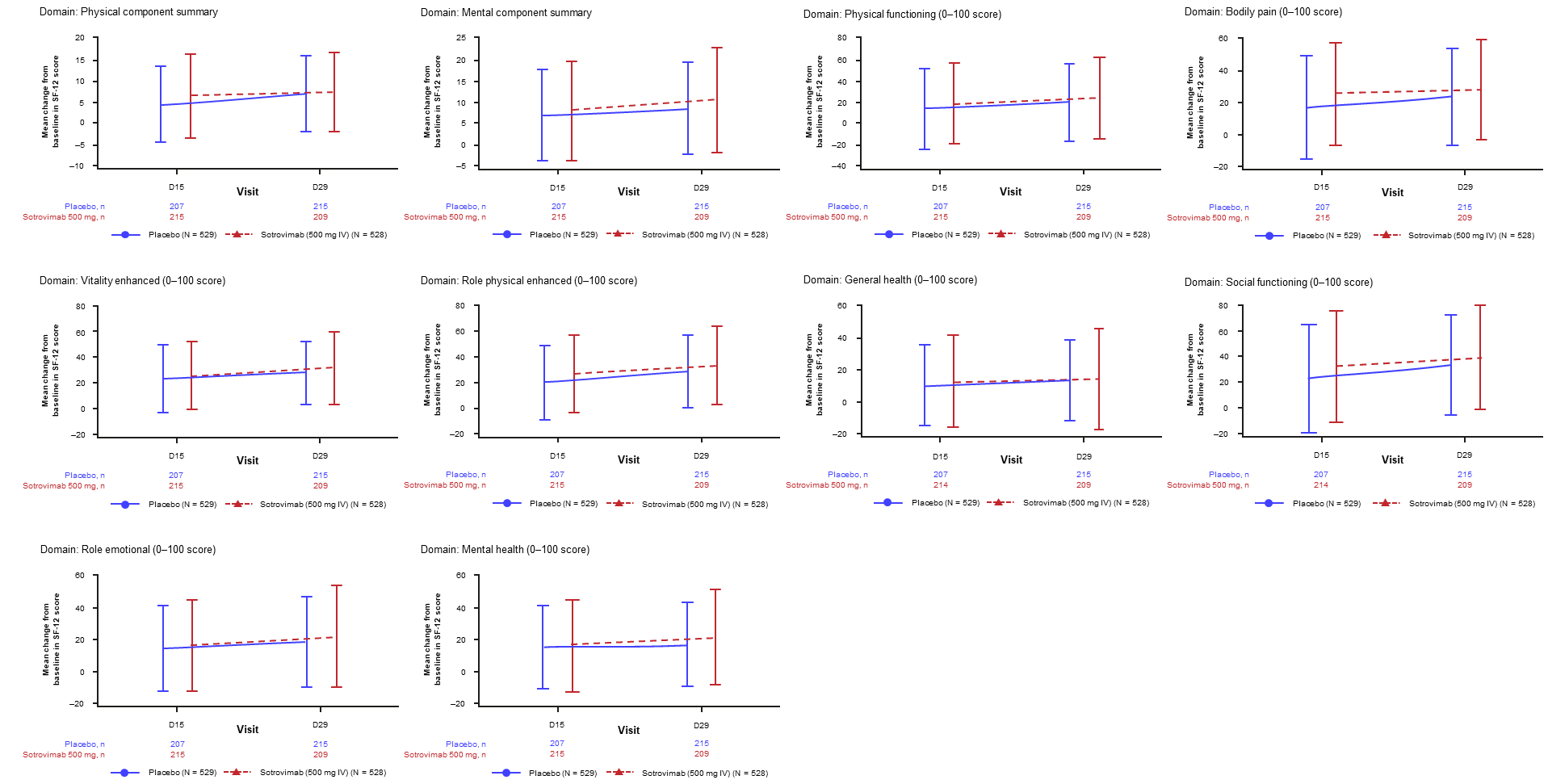


*D* day, *IV* intravenous, *SD* standard deviation, *SF-12* Short Form-12
